# Age-dependent differential iron deficiency responses of rosette leaves during reproductive stages in *Arabidopsis thaliana*

**DOI:** 10.1093/jxb/eraf207

**Published:** 2025-05-21

**Authors:** Mary Ngigi, Mather Khan, Ricarda Remus, Shishir K Gupta, Petra Bauer

**Affiliations:** Institute of Botany, Heinrich-Heine-University, D-40225 Düsseldorf, Germany; Cluster of Excellence on Plant Science (CEPLAS), Heinrich-Heine-University, D-40225 Düsseldorf, Germany; Institute of Botany, Heinrich-Heine-University, D-40225 Düsseldorf, Germany; Cluster of Excellence on Plant Science (CEPLAS), Heinrich-Heine-University, D-40225 Düsseldorf, Germany; Division of Plant Science & Technology, C.S. Bond Life Sciences Center, University of Missouri, Columbia, MO 65211, USA; Institute of Botany, Heinrich-Heine-University, D-40225 Düsseldorf, Germany; Institute of Botany, Heinrich-Heine-University, D-40225 Düsseldorf, Germany; Cluster of Excellence on Plant Science (CEPLAS), Heinrich-Heine-University, D-40225 Düsseldorf, Germany; Institute of Botany, Heinrich-Heine-University, D-40225 Düsseldorf, Germany; Cluster of Excellence on Plant Science (CEPLAS), Heinrich-Heine-University, D-40225 Düsseldorf, Germany; Lawrence Berkeley National Laboratory, USA

**Keywords:** Copper, gene co-expression, iron, leaf signaling, manganese, old leaves, reproduction, rosette leaves, young leaves, zinc

## Abstract

Iron (Fe) is essential for plant development throughout the life cycle. Rosette leaves are responsive to Fe supply in *Arabidopsis thaliana*. Little is known about the dynamics of Fe deficiency (−Fe) responses of rosette leaves during the reproductive stages. We studied the dynamics of Fe-dependent responses at four consecutive reproductive stages (rosette, bolting, flowering, and mature silique stages, herein named RS, BS, FS, and MS). We examined the growth of rosette leaves, elemental contents, and gene expression patterns of Fe homeostasis genes belonging to differently regulated groups. We determined individual leaf sizes during 7 d of +Fe and −Fe treatment at the RS. Young leaves responded to −Fe with growth inhibition and yellowing. Old and young leaves differed in gene expression patterns and elemental contents. Differences were noted between the early and late reproductive stages (primarily RS and BS versus MS), and correlations between ionomic contents and gene expression were detected. All leaves had induced Fe recycling genes under −Fe. Our findings highlight a developmental stage-dependent modulation of +Fe and −Fe responses in leaves. We discuss possible leaf signaling mechanisms accounting for the distinct responses between old and young leaves. This insight is informative to strengthen our understanding of plant Fe management.

## Teaser

This study explores reproductive stage-dependent responses of rosette leaves to iron availability, revealing distinct growth, elemental contents, and gene expression patterns between old and young leaves and stages.

## Introduction

Iron (Fe) is a vital micronutrient for all organisms. In plants, Fe is central for the many fundamental Fe-requiring metabolic processes such as photosynthesis, nutrient assimilation, chlorophyll synthesis, and oxidative stress defense (Briat *et al*., 2007; Jeong and Guerinot, 2009). Fe is often not bioavailable in soil and is limiting during plant growth, requiring acclimation of plant Fe physiology. The complexities of Fe homeostasis and networks of Fe-related cell physiological functions can be unraveled by exposing plants to Fe deficiency (−Fe) and recording growth data, ionomics profiles, and Fe-related gene expression patterns. Understanding Fe-regulatory pathways paves the way for innovations in translational plant biotechnology, such as to generate biofortified Fe-rich crops for food nutritional security that are suited for sustainable agroecological practices.

*Arabidopsis thaliana* has served widely as a model species to generate knowledge about the regulation and genetic networks that steer the ways in which Fe is acquired and allocated in plants (Sági-Kazár *et al*., 2022). Yet, there are some gaps in our understanding. Many studies focused on investigating the roles of Fe-regulatory genes, particularly in young seedlings and during the vegetative growth phase (Mai *et al*., 2016; Khan *et al*., 2018; Muhammad *et al*., 2022; Nguyen *et al*., 2022; Pu and Liang, 2023). The few studies that examined Fe management during reproduction concluded that *de novo* Fe uptake and Fe recycling are important to compensate for a lack of Fe, and that rosette leaves may act as Fe reserves that possibly supply Fe to newly developing organs through remobilization mechanisms (Waters and Grusak, 2008; Schuler *et al*., 2012; Pottier *et al*., 2019). Yet, to our knowledge, a detailed and systematic study that investigates how different rosette leaves of Arabidopsis respond to −Fe cues during the early and late reproductive stages is missing.

During the reproductive stages, the rosette leaves can be classified into old and young leaves, distinguishing them based on their age whereby first true leaves are fully matured at the onset of reproduction, while the later developed leaves continuously grow and expand until late reproductive stages. Old and young leaf types differ in morphology and size, and hints are available that they also show variation in the regulation of Fe homeostasis under changing nutrient conditions (Schuler *et al*., 2012; Nguyen *et al*., 2022; Swartz *et al*., 2023). Better understanding of how different types of leaves manage Fe and Fe-related gene expression will provide new insights into leaf Fe signaling in Arabidopsis. Numerous mutants and tools are available in *A. thaliana* that can be leveraged for further studies to better understand the genetic mechanisms directing Fe physiology during reproduction.

Gene co-expression analysis is a powerful tool to decipher Fe-regulatory networks, cell functions, and pathways for Fe management under Fe sufficiency (+Fe) and Fe deficiency (−Fe) conditions (Ivanov *et al*., 2012; Schwarz and Bauer, 2020). Co-expression indicates that different genes interact so that their collective expression leads to specific cellular and physiological outcomes being controlled by similar stimuli such as +Fe or −Fe. Of particular interest, the similar expression patterns of co-expressed −Fe-regulated genes across different conditions, time points, or tissues indicate that they are most likely to be regulated by the same group of transcription factors and involved in related Fe-regulatory processes (Schwarz and Bauer, 2020; Spielmann *et al*., 2023). Analyzing −Fe-induced co-expressed genes has helped to understand the complexities of Fe-regulatory systems. The Fe deficiency-induced co-expressed genes are divided into subcategories of co-expression and co-regulation networks, that reflect the division into different Fe-related functions (Ivanov *et al*., 2012; Schwarz and Bauer, 2020). One subset of genes or co-expression cluster induced in response to −Fe in seedling roots serves to mobilize and acquire *de novo* Fe from soil, a process relevant in the root epidermis. This subset is also referred to as ‘FIT-dependent’ since its up-regulation requires the presence of a transcription factor called FIT (FER-LIKE FE DEFICIENCY-INDUCED TRANSCRIPTION FACTOR). A second subset is often termed ‘FIT-independent’ since the expression of the genes in this cluster is particularly high when FIT function is lost, leading to the inability to acquire *de novo* Fe and hence induction of −Fe signals in the plant. This subset of co-expressed genes is active in both roots and shoots, and functions to mobilize and allocate internal Fe from root to shoot or within the shoot, and this subset is primarily expressed in the root epidermis, the root stele, and the leaf vasculature (Wang *et al*., 2007; Stacey *et al*., 2008; Long *et al*., 2010; Selote *et al*., 2015). This co-expression cluster encodes proteins that recycle Fe from vacuolar stores, control intercellular Fe redistribution through phloem-based Fe–nicotianamine chelates, unload towards Fe sinks (Lanquar *et al*., 2005; Schuler *et al*., 2012; Bastow *et al*., 2018), and sequester nicotianamine in root vacuoles for the management of metal ions (Haydon and Cobbett, 2007; Haydon *et al*., 2012). The concept of distinguishing co-expressed ‘FIT-dependent’ and ‘FIT-independent’ genes has been applied to classify the hierarchy of Fe responses and decipher signaling and regulatory events (Ivanov *et al*., 2012; Schwarz and Bauer, 2020; Lichtblau *et al*., 2022; Muhammad *et al*., 2022).

We here set out to investigate and correlate the −Fe responses of old and young rosette leaves in a spatial- and time-resolved manner across four reproductive stages. We recorded growth dynamics, Fe-related gene expression changes, and metal ion contents under +Fe and −Fe exposure. We hypothesized that reproductive transition profoundly impacts Fe homeostasis, and we predicted an increase in −Fe sensitivity in old and young leaves from early to late reproductive stages. However, this systematic study uncovered unexpected dynamics of reproductive and stage-specific Fe homeostasis regulation under changing Fe supply, with distinct patterns in old and young leaves.

## Materials and methods

### Plant materials and growth conditions

**Fig. 1.**
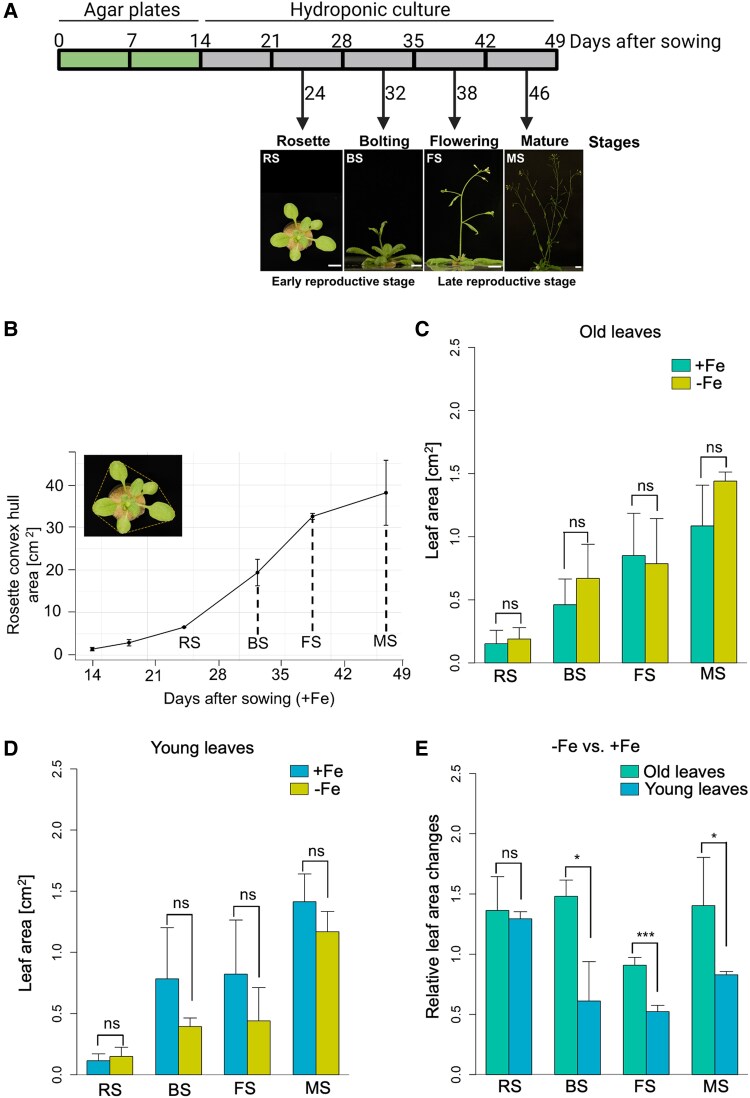
Growth dynamics during progression of the Arabidopsis life cycle. (A) Overview of the experimental set up and reproductive growth stages selected for analysis of *Arabidopsis thaliana*. Seedlings were germinated on Hoagland agar plates before transplanting to standard hydroponic medium with 25 µM Fe (+Fe) for growth to the four selected stages under long-day conditions: rosette stage (RS), bolting stage (BS), flowering stage (FS), and mature silique stage (MS) before further analysis; scale bar = 1 cm. Further details on the reproductive growth stages are given in Supplementary Fig. S1A, B. (B) Rosette convex hull area increase of plants continuously grown on medium with +Fe until the mature stage. The area included all leaves forming around the canopy plus their petioles (*n* = 2–8). (C and D) Absolute leaf areas of old and young leaves under +Fe and −Fe, respectively. Plants were transferred to +Fe and −Fe conditions for 3 d at the indicated growth stages. Old and young leaves harvested for analysis are described in Supplementary Fig. S1C. (E) Relative changes in leaf area of old and young leaves under −Fe compared with +Fe conditions at RS to MS. Data in (C and D) represent average values of biological replicates (*n* = 5). Error bars show ±SD. Statistical analysis was done by Student's *t*-test (***P < 0.001, *P < 0.05, ‘ns’ *P* > 0.05). The plant growth scheme was created in Biorender.

*Arabidopsis thaliana* ecotype Columbia (Col-0) seeds were surface-sterilized, stratified for 48 h at 4 °C in the dark, and grown vertically on half-strength Hoagland agar plates (Lichtblau *et al*., 2022) in a growth chamber (CLF Plant Climatics, Wertingen, Germany) at a 16 h/8 h photoperiod with 100 μmol m^−2^ s^−1^ light intensity, 22 °C day/20 °C night, and 50–60% relative humidity. Twelve-day-old seedlings were transferred into a hydroponic set up as previously described by Nguyen *et al*. (2016) using a quarter-strength Hoagland hydroponic medium containing macronutrients [0.375 mM MgSO_4_, 0.75 mM Ca (NO_3_)_2_, 0.25 mM KH_2_PO_4_, 0.625 mM KNO_3_] and micronutrients [5 μM MnSO_4_, 1 μM ZnSO_4_, 0.75 μM CuSO_4_, 0.0375 μM (NH_4_)_6_Mo_7_O_24_, 25 μM KCl, and 25 μM H_3_BO_3_]. FeNaEDTA at 25 µM was added to the nutrient medium as sufficient Fe (+Fe). The medium was replaced every 3 d with freshly prepared hydroponic solution aerated using air pumps. Plants were grown up to four reproductive stages as previously described in Boyes *et al*. (2001), herein named the rosette stage (RS; early reproductive stage with a <1 cm inflorescence stem and closed inflorescence bud, 24 d old), bolting stage (BS; inflorescence stem 2–4 cm with a closed inflorescence bud, advanced inflorescence with beginning of internode elongation, 32 d old), flowering stage (FS; main inflorescence stem >5 cm with 3–5 open floral buds, 38 d old), and mature silique stage (MS; main inflorescence stem >8 cm with 6–7 mature yellowing siliques, 46 d old) (Fig. 1A; Supplementary Fig. S1A, B). At the selected stages, half of the plants were transferred to +Fe, and the other half were exposed to −Fe. Fe treatments were started 3–4 h after the growth chamber lights had been switched on. −Fe hydroponic solution was the same quarter-strength Hoagland medium described above, with no Fe supplementation (0 µM FeNaEDTA). After 3 d of +Fe and −Fe treatment, roots, old leaves, young leaves, and shoot apices were excised. Leaves were pooled according to the scheme shown in Supplementary Fig. S1C for the different growth stages. For leaf-by-leaf analysis, 3-week-old hydroponically grown plants were transferred at the RS to the +Fe or −Fe condition, and each day leaves were harvested from day 1 to day 7 for leaf area analysis.

### Determination of rosette and leaf size

Plant rosette and leaf images were taken from the top using a Sony Alpha 6000 digital camera. Images were imported into ImageJ version 1.8.0 (https://imagej.net/ij/) for further processing and analysis. In brief, rosette images were converted to black and white, and a color threshold was set before quantification of rosette convex hull area. This was calculated from the surface area covered by the circumference of the longest leaves as shown in Fig. 1B. Individual leaves were dissected from each plant and arranged in a row from oldest to youngest leaf as illustrated in Supplementary Fig. S1C. Leaf areas were determined by the area corresponding to that of the leaf blades.

### Scanning electron microscopy

Hand-dissected shoot apices were fixed overnight in 1% paraformaldehyde solution then dehydrated in a series of 30, 50, 70, and 90% ethanol, each for 20 min, and 100% ethanol for 40 min. Samples were completely dehydrated to a critical point in a dryer (EMCPD 300, Leica) at 70 bar with 13 °C/40 °C cool–heat cycles for 3 h. Samples were gold-coated under a high vacuum sputter (CCU-010, Safematic) before imaging with an electron microscope (Supra 40VP, Zeiss). Images were analyzed in ImageJ v 1.8.0.

### Gene expression analysis by reverse transcription–quantitative PCR

All steps of reverse transcription–quantitative PCR (RT–qPCR) analysis were conducted according to Ngigi and Bauer (2023). Briefly, plant materials were harvested and immediately deep-frozen in liquid nitrogen. Hand-dissected inflorescence shoot apices, however, were first placed in RNAlater (Thermofisher Scientific) and then deep-frozen. Samples were pulverized in a Precellys 24 tissue homogenizer (Peqlab, Germany). Total RNA extraction from all plant samples was performed using a plant RNA kit (Qiagen, Germany). Approximately 1 µg of RNA was treated with DNase I (Thermofisher Scientific) for removal of genomic DNA contamination followed by first-strand cDNA synthesis using the Revert-aid reverse transcriptase kit (Thermofisher Scientific). Real-time qPCR was performed using 2× iTaq SYBR Green Supermix detection (Bio-Rad) on C1000 Touch CFX384 (Bio-Rad). qPCR data were individually checked and validated by melt curve analysis. Mean absolute normalized transcript abundances were calculated thanks to mass standard analysis and referral to the expression of the reference gene *EF1B-ALPHA2*. qPCR primer sequences are listed in Supplementary Table S1.

### Ionomics analysis

Roots and leaves were collected. Residual Fe in the root apoplast was removed by washing the roots in 20 mM Tris, 5 mM EDTA, pH 8 for 5 min before rinsing in deionized water. Old and young leaves were washed in deionized water to remove Fe on the leaf surface. All samples were dried at 65 °C for 3 d (or longer as needed) before grinding to a fine powder. Ground material was transferred into metal-free 5 ml screw-cap tubes (Eppendorf, Germany, Cat. No. 0030122305) and 1 ml of 67% nitric acid was added. Samples were digested by boiling until a clear liquid was obtained. Elemental analysis was performed using inductively coupled plasma-mass spectroscopy (ICP-MS, Agilent 7700). A total of five plants were pooled together for material collection per sample in three independent experiments. Elemental concentrations were determined with the help of mass standards and normalized to sample dry weights.

### Statistical analysis and data visualization

Data were analyzed by one-way ANOVA with Tukey’s Honest Significant Difference (HSD) test for multiple comparisons and Student’s *t*-test for pairwise comparisons. Bar graphs and line charts show the mean and SD of 3–5 biological replicates. Means that were statistically significantly different are indicated by different letters (*P* < 0.05). Correlation analysis of normalized gene expression values and mineral content was done using Pearson’s correlation with significance *P-*value < 0.05. Dynamic correlation networks were visualized with the assistance of Julius AI (https://julius.ai/). Heatmaps were generated using mean absolute normalized gene expression values. The data were processed using R (version 4.4.1). Gene names were assigned as row labels. *Z*-score normalization was applied to each gene (row-wise) to account for variability in baseline expression. The *Z*-scores were calculated using the scale () function in R, with the following formula:


$$Z=\frac{X-\mu }{\sigma }$$

where *X* is the expression for a specific sample, μ is the mean expression of the gene across all samples, and σ is the SD of the gene expression. This row-wise *Z*-score normalization made it possible to compare expression levels of each gene across tissues under both +Fe and −Fe, allowing positive *Z*-scores to indicate above-average expression for high expression values and negative *Z*-scores to indicate below-average expression for low expression values. Unbiased gene clustering analysis was done by hierarchical clustering. The heatmaps were generated using the pheatmap package in R.

## Results

### The size of the leaf rosette increases during four reproductive phases

To assess plant responses during the reproductive phase, Arabidopsis wild-type (Col-0) plants were grown for up to 7 weeks in sufficient Fe conditions (+Fe) in a hydroponic system. Four meaningful reproductive stages were selected for plant phenotyping (Fig. 1A). At the rosette stage (RS, 24 d) plants did not yet have an emerged inflorescence stem, the rosette had ∼11 emerged leaves, and plants had closed inflorescence buds (Fig. 1A; Supplementary Fig. S1A–D). At the bolting stage (BS, 32 d), the main inflorescence stem had emerged and was between 2 cm and 4 cm tall, and the rosette had ∼11 examinable leaves which had grown bigger than at the RS, with closed floral buds. At the flowering stage (FS, 38 d), the main inflorescence stem had elongated and was taller than 5 cm with 3–5 open flowers; the rosette had 13–17 examinable leaves. At the mature silique stage (MS, 46 d), more than three lateral inflorescence branches had emerged, the main inflorescence stem was longer than 8 cm with 6–7 drying siliques, each being longer than 1 cm, and the rosette had > 20 examinable leaves (Fig. 1A; Supplementary Fig. S1A–D). Rosette convex hull area is a commonly used parameter for growth evaluation of *A. thaliana* (Saini *et al*., 2022). The rosette growth curve was rather steep (Fig. 1B). Maximal rosette dimensions were reached at the MS. The fastest rosette size increase took place between the RS and FS, particularly around BS, while it slowed down before the RS and between the FS and MS (Fig. 1B). We suspected that Fe was needed for rosette leaf growth to occur during the reproductive stages, particularly during the rapid size increase.

### Leaf areas of young leaves were reduced in response to –Fe versus +Fe

We tested whether leaf growth was linked to Fe availability. According to a previous study, we termed the fully grown leaves that had reached their final or nearly final size ‘old leaves’, while the still developing leaves were termed ‘young leaves’ (Schuler *et al*., 2012). It is reported that the sizes of young leaves change upon micronutrient deficiency (Swartz *et al*., 2023). However, whether rosette leaf growth is still influenced at the reproductive growth stages is not yet known. We grew plants up to the four selected reproductive stages and exposed them to +Fe and −Fe for 3 d. Individual leaves were dissected (Supplementary Fig. S1C) and leaf areas measured, then grouped into old and young leaves taking into account the leaf characteristics as described in Swartz *et al*. (2023) (Supplementary Fig. S1C). There were no significant differences between either old leaves or young leaves under +Fe and −Fe, respectively (Fig. 1C, D). At the RS, old and young leaves had similar sizes when plants were exposed to +Fe and −Fe treatments (Fig. 1D), presumably because they were still small and not expanding as much as during the BS to MS. However, when comparing the relative leaf area changes between −Fe and +Fe, there were differences between old and young leaves at BS, FS, and MS (Fig. 1E), which can be attributed to an age-dependent expansion during reproductive development.

To assess whether overall plant growth was also affected by −Fe treatment, we collected biomass data. The root FWs had increased steadily from RS to MS. At the RS and BS, there were no significant differences in root FWs between +Fe and −Fe treatment; however, at the FS and MS, there was a drastic reduction of root FW after the 3 d −Fe exposure compared with +Fe (Supplementary Fig. S1E). This finding potentially represents a survival strategy whereby root growth at late reproductive stages may slow down under Fe-limiting conditions due to Fe release in cells, causing Fe toxicity (Naranjo-Arcos *et al*., 2017). Shoot biomass increased steadily from RS to MS. Particularly between FS and MS, shoot FWs had drastically increased, but there was no difference following the 3 d +Fe and −Fe conditions (Supplementary Fig. S1F). The shoot biomass increase was caused by the growing inflorescence stems, flowers, and siliques, and perhaps the growth differences could not be detected by total weight.

Taken together, our growth system is suited to detect Fe-dependent growth dynamics in the rosette during the reproductive growth phases. In particular, the rapid increase of the sizes of young leaves and the rosette visible at the BS is influenced by Fe supply and suppressed in response to −Fe. Since the responses of rosette leaves during the reproductive phase are underexplored, we focused on rosette leaf physiology in the continuation of this study.

### Leaf-by-leaf analysis during consecutive days revealed a rapid growth arrest of young leaves in response to –Fe

**Fig. 2.**
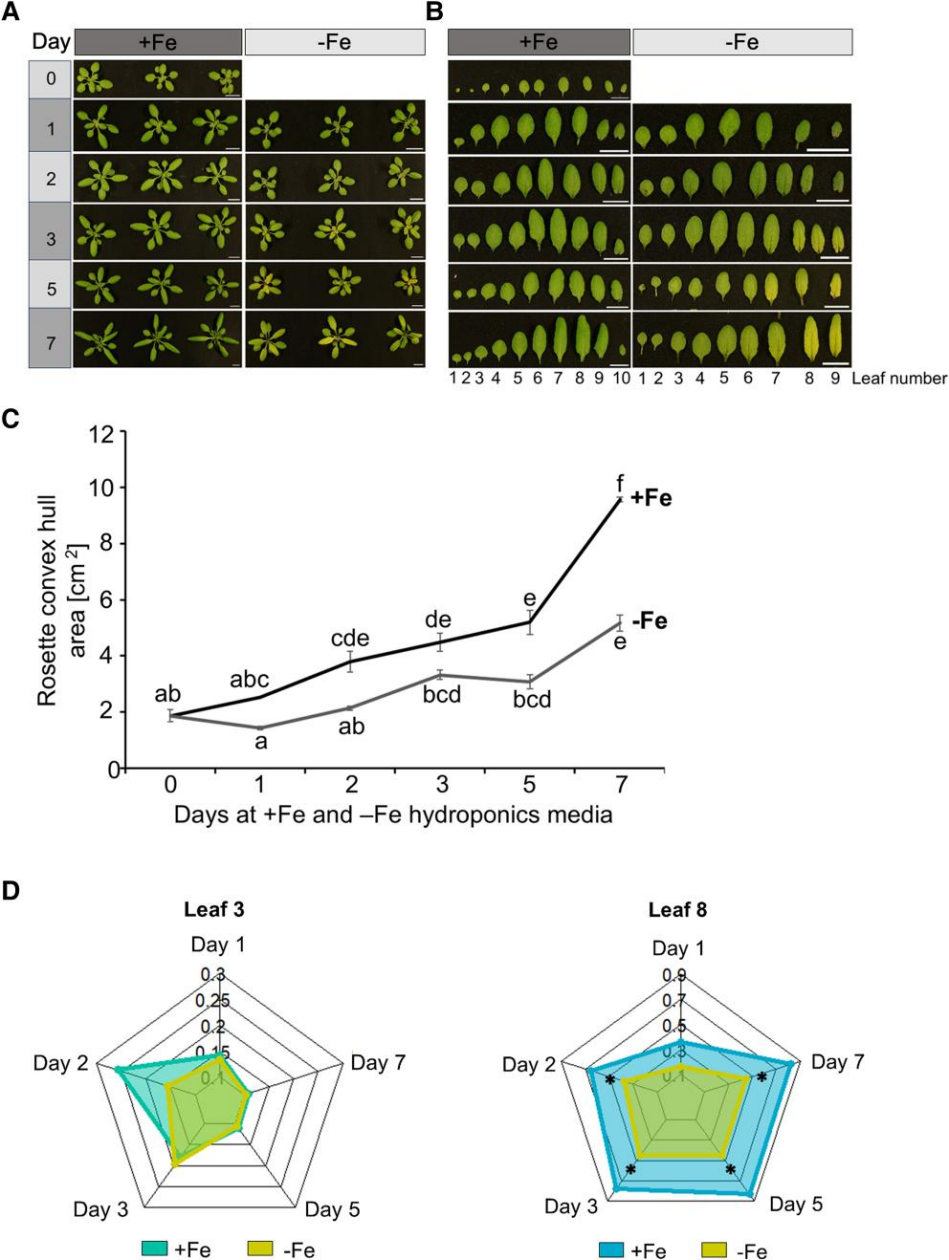
Leaf-by-leaf analysis of *Arabidopsis thaliana* at the rosette stage (RS). (A) At the RS, plants were transferred to respective Fe-sufficient (+Fe) or -deficient (−Fe) Hoagland media solutions and growth was recorded from day 0 till day 7. (B) Leaf images of leaf 1 (L1) to 10 (L10) under the two Fe conditions, as indicated. (C) Cumulative rosette convex hull area as recorded from (A). Data represent means of biological replicates (*n* = 3). Error bars represent ± SD. Statistical analysis was done using one-way ANOVA and Tukey’s HSD test. Means followed by the same letter are not significantly different (*P* < 0.05). (D) Radial charts showing the leaf areas of leaf 3 and 8 after exposure to either +Fe (control) or −Fe conditions from day 1 to day 7. Radial charts were plotted in R Studio using the ‘fmsb’ package. Plants were grown for 3 weeks before culturing in either +Fe or −Fe conditions. Asterisks show where leaf areas were statistically significantly different between +Fe and −Fe conditions, (*P* < 0.05), one-way ANOVA and Tukey’s HSD test.

To better resolve the Fe-dependent influence on leaf growth, we selected the RS stage and exposed plants to +Fe and −Fe. The rosette convex hull area and leaf areas of up to the first 10 leaves amenable to examination were determined on days 1, 2, 3, 5, and 7 (Fig. 2). Between days 5 and 7, the rosette sizes of +Fe plants began to increase rapidly and, at these time points, significant differences became apparent between +Fe- and −Fe-grown plants (Fig. 2A–C). In the +Fe condition, rosette leaves displayed a green color throughout the 7 d period, while under −Fe conditions the visual symptoms of leaf chlorosis became evident on day 2 and intensified as the experiment progressed (Fig. 2A, B). Notably, −Fe leaf chlorosis was more pronounced in the young leaves, such as L8–L10, compared with old leaves, such as L3–L5 (Fig. 2B). The young leaves (e.g. L8) were smaller under −Fe than +Fe from day 1 to day 7 (Fig. 2D), similar to L5–L7 (Supplementary Fig. S2D–F). L3, an old leaf, did not show changes in the leaf areas on day 1 and days 5–7 irrespective of treatments, showing that they had reached their final size at the RS. However, L5–L8 increased in size from day 1 until day 7, indicating that they were continuously developing young leaves (Fig. 2D; Supplementary Fig. S2D–F). Interestingly, already after 1 d of −Fe treatment, the leaf areas were smaller in all these four leaves (L5–L8) compared with +Fe (Fig. 2D; Supplementary Fig. S2D–F). This shows that −Fe affected leaf growth and expansion immediately within a day.

Taken together, these findings underscore the critical role of Fe for the growth of young leaves. Young leaves were more susceptible to developing leaf chlorosis than old leaves. The differential phenotypic responses observed between old and young leaves speak in favor of differential Fe homeostasis physiology between old and young leaves. The very rapid growth response indicates that −Fe sensing and signaling directly control leaf growth.

### Expression of Fe-regulated genes points to Fe recycling taking place in old leaves at the rosette and bolting stages

**Fig. 3.**
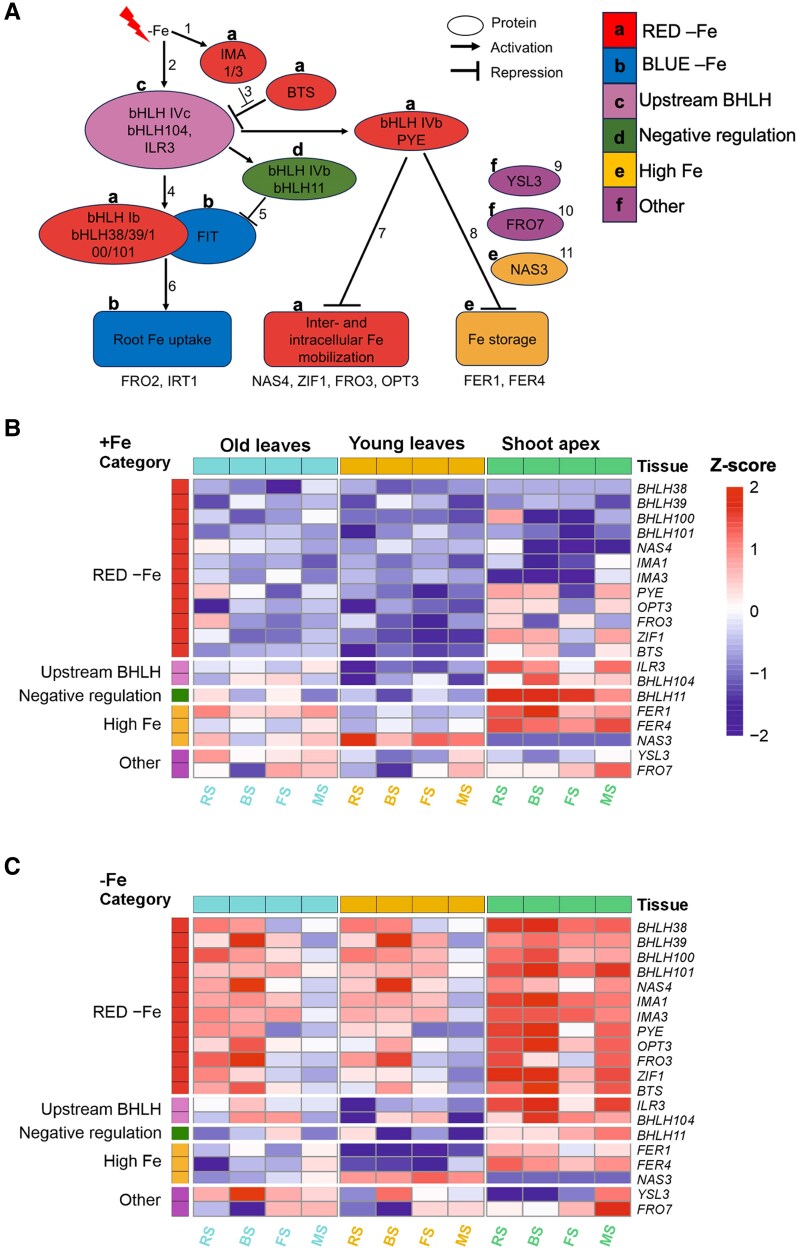
Gene expression analysis of selected Fe homeostasis genes. (A) Schematic illustration showing connections and functions of Fe homeostasis genes used in gene expression analysis. Represented is the Fe-regulated transcriptional cascade and multiple layers of co-expressed genes involved in plant Fe homeostasis. Exemplary references: ^1^(Kobayashi *et al*., 2021), ^2^(Zhang *et al*., 2015), ^3^(Lichtblau *et al*., 2022), ^4^(Li *et al*., 2016 ), ^5^(Tanabe *et al*., 2019), ^6^(Trofimov *et al*., 2019), ^7^(Long *et al*., 2010), ^8^(Tissot *et al*., 2019), ^9^(Chu *et al*., 2010), ^10^(Mukherjee *et al*., 2006), ^11^(Schuler *et al*., 2012). Letters above the genes correspond to the co-expressed gene categories. (B and C) Heatmaps showing the absolute expression profiles of 20 selected Fe homeostasis genes in old leaves, young leaves, and inflorescence shoot apex after exposure to +Fe and −Fe for 3 d. Plants were grown to the various stages, as indicated, before transfer to either Fe solutions. The growth stages RS, BS, FS, and MS correspond to rosette stage, bolting stage, flowering stage, and mature stage, respectively. Heatmaps were generated in R studio version 4.4.1 using calculated *Z*-scores of normalized gene expression values of each gene across tissues for both +Fe and −Fe. Functional gene category annotations are displayed on the left side of the heatmap, with tissue type annotations displayed on the top. Gene up- and down-regulation are colored according to their *Z*-scores. *P* < 0.05 (*n* = 3). The color scale ranges from 2 to −2 to represent the variation in gene expression.

To capture differential regulation patterns of leaves across the reproductive stages, we studied the gene expression patterns of 23 Fe homeostasis genes by analyzing transcript amounts using RT–qPCR, that corresponded to meaningful lists of genes from previously reported studies (Mai *et al*., 2016; Schwarz and Bauer, 2020). We grew plants as above and collected old and young rosette leaves of +Fe and −Fe plants across the four reproductive stages to investigate gene expression in a space- and time-resolved manner (old leaves were L3–L5 from RS to FS, and L5–L7 at MS, as L1–L4 had senesced at this stage; young leaves were L9–L11 from RS to FS, and L14–L17 at MS; Supplementary Fig. S1C). Inflorescence shoot apices were collected since they harbored the youngest leaves and leaf primordia. Roots were harvested to estimate the Fe status of plants. The 23 analyzed genes were grouped into six categories or clusters according to their previously described co-regulation and expression patterns, representing different roles in Fe homeostasis (Fig. 3A).

The gene expression data were remarkable in several ways. At first, we investigated gene expression in the leaf and shoot apex samples. To get an overview of how the genes are expressed across the four stages and tissues, we performed a principal component analysis (PCA) of all genes. Results revealed that the FIT-independent gene cluster, herein named the ‘RED –Fe cluster’, separately grouped from other gene categories, highlighting differential regulation (Supplementary Fig. S3). The RED –Fe cluster genes which serve the recycling of Fe in the whole plant were overall expressed more under −Fe than under +Fe in both the old and young leaves and the shoot apex samples (Fig. 3B, C). In the leaf samples, this was particularly evident at the early reproductive stages RS and BS where all 12 genes in the RED −Fe cluster were expressed at a higher level than at the late reproductive stage, MS (Fig. 3C). We see this observation as a hint that the –Fe-induced RED cluster genes do not need to be expressed strongly in the rosette leaves at the late reproductive stages when siliques are developing. We suspect that at FS and MS the rosette leaves become less susceptible to −Fe signals than at earlier stages. Surprisingly, gene expression of the RED –Fe cluster remained induced in the inflorescence shoot apices at FS and MS, which provided a hint that these genes were regulated by Fe homeostasis in the reproductive shoot apical meristem. We noted another interesting feature about the expression patterns of RED –Fe cluster genes, namely that their co-expression was split in the +Fe samples. The four basic helix–loop–helix (*BHLH*) subgroup Ib genes (*BHLH038*, *BHLH039*, *BHLH100*, and *BHLH101*), and *NAS4*, *IMA1*, and *IMA3* genes were expressed at comparatively lower levels under +Fe than *PYE*, *OPT3*, *FRO3*, *ZIF1*, and *BTS*, which were more highly expressed under +Fe and even quite elevated in the +Fe shoot apices. Hierarchical clustering revealed that RED –Fe genes were differentially up-regulated and co-expressed by −Fe, suggesting co-regulation by Fe deficiency in shoot samples (Supplementary Fig. S4). In sum, the −Fe regulation of RED –Fe cluster genes is most important in both old and young leaves, in particular at BS and RS, as well as in the inflorescence shoot apices at all reproductive stages. There is evidence that *PYE*, *OPT3*, *FRO3*, *ZIF1*, and *BTS* are differently expressed from other RED −Fe cluster genes, which can hint at different upstream regulation or functions.

The next interesting observation was that the expression of genes encoding the positive regulators of RED −Fe cluster genes, namely *ILR3* and *BHLH104*, summarized as Upstream BHLH, and the negative regulator, namely *BHLH11*, was consistent with that of the RED –Fe cluster genes *PYE*, *OPT3*, *FRO3*, *ZIF1*, and *BTS*. When these RED –Fe cluster genes were up-regulated specifically at BS under −Fe, it was also the case for the positive regulators *ILR3* and *BHLH104*, while the negative regulator *BHLH11* was down-regulated, especially in the old leaves. The upstream regulator genes were not Fe regulated and were expressed at BS to MS in old and young leaves, and in all the stages in inflorescence shoot apices (Fig. 3B, C). For *ILR3* and *BHLH104*, this was expected, but for *BHLH11* it was unexpected, since it was reported that BHLH11 is down-regulated by −Fe (Tanabe *et al*., 2019). With respect to a possible difference of expression in old versus young leaves, we noted a tendency that under −Fe, expression was higher in old versus young leaves in agreement with the expression of target RED –Fe cluster genes (Fig. 3C).

Another remarkable point was that the expression levels of genes up-regulated at Fe sufficiency and excess conditions, herein named the ‘high Fe’ gene cluster, such as the *FERRITIN* gene *FER1* (Briat *et al*., 2010; Roschzttardtz *et al*., 2013), remained relatively more highly expressed throughout the stages under +Fe in the old leaves compared with −Fe and young leaves (Fig. 3B). This was not the case for *FER4* expression. On the other hand, *FER1* and *FER4* showed similar high expression in the inflorescence shoot apex samples across the stages under +Fe, with slight down-regulation under −Fe (Fig. 3B, C). Surprisingly, *NAS3* expression levels were consistently up-regulated in the young leaves at all the four reproductive stages in both Fe conditions, but down-regulated in shoot apices (Fig. 3B, C). With respect to the question about differences in the leaf samples under −Fe, we noted tendencies of higher expression of the high Fe genes *FER1* and *FER4* in old versus young leaves, both of which were significant at MS, whereas *NAS3* was more highly expressed in young versus old leaves across the four stages (Fig. 3C). Thus, the high Fe gene cluster expression did not distinguish the four reproductive stages, but it might be a marker for Fe-regulated processes in the old versus young leaves and in the shoot apex. Perhaps *NAS3* plays a major role in Fe remobilization and export.

Finally, *YSL3* was expressed at higher levels in leaves than in the shoot apex samples, and in some case there appeared to be up-regulation under −Fe versus +Fe, such as at the BS in leaves (Fig. 3B, C). *FRO7* had similar expression between +Fe and −Fe, and was mostly expressed at higher levels in shoot apex samples than in leaves, especially at MS (Fig. 3B, C). Very interestingly, the expression of *YSL3* was up-regulated in old versus young leaves under −Fe at all the four stages. *FRO7* expression differences were not noted between leaves and Fe supply (Fig. 3B, C).

The root gene expression patterns were also very remarkable. As compared with rosette leaves and shoot apex samples, the expression levels were surprisingly lower for most genes analyzed above (Supplementary Fig. S5). Although the FIT-dependent genes, herein named the BLUE –Fe cluster, and previously known as root −Fe markers, were on the one hand expressed at similar or higher levels in roots versus the shoot organs, but on the other hand quite strongly expressed at BS in the leaf samples, and at MS in the shoot apex samples. Perhaps the root system is either no longer very sensitive to −Fe or it also consists of different zones that respond differently to −Fe.

Taken together, the leaf and shoot apex gene expression patterns were rather complex during the reproductive stages. There were differences in expression between +Fe and −Fe, between the four reproductive stages, and between old and young leaves, as well as the inflorescence shoot apex. Two major conclusions could be drawn. Fe recycling genes of the RED −Fe cluster were mostly expressed at the RS and BS stages in old and young leaves under −Fe, while high Fe markers were rather uniformly expressed throughout all stages under both +Fe and −Fe. Most genes that discriminated against old and young leaves were expressed at higher levels in old versus young leaves, especially the RED −Fe cluster genes *PYE*, *OPT3*, *FRO3*, *ZIF1*, *BTS*, and *YSL3* under −Fe, as well as *FER1* under +Fe and in the high Fe genes cluster. Only *NAS3* was found to be consistently more highly expressed in young than old leaves and shoot apices. These findings indicate that different physiological states are associated with the organs at the different reproductive stages.

### Mineral contents differ between old and young leaves at the early reproductive stages

**Fig. 4.**
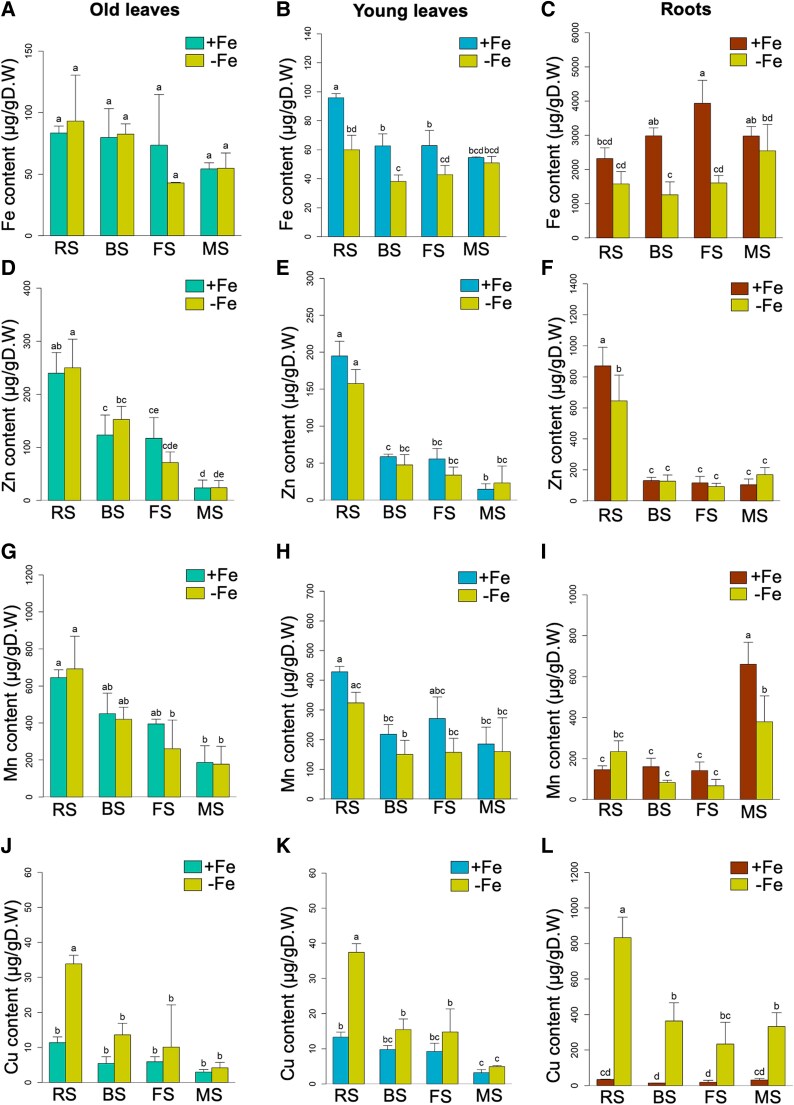
Ionomic analysis of leaves and roots. Fe (A–C), Zn (D–F), Mn (G–I), and Cu (J–L) content per DW in old leaves, young leaves, and roots of plants grown at 25 µM Fe (+Fe) or 0 µM Fe (−Fe) conditions and at the four growth stages; RS, BS, FS, and MS. Plants were exposed to 3 d of +Fe or −Fe before sample collection. Data represent the mean of three biological replicates (*n* = 3). Error bars represent the ± SD. Letters on top of each bar show the level of significance according to one-way ANOVA and Tukey’s HSD test, *P* < 0.05.

To test whether old and young leaves may be subject to different Fe and metal ion physiology, we determined the elemental contents of Fe, Zn, Mn, and Cu. First, we compared the elemental contents between +Fe and −Fe separately for old and young leaves and roots (Fig. 4). In old and young leaves, there was a clear trend for metal contents to decrease from RS to MS in the +Fe and −Fe conditions (Fig. 4A, B, D, E, G, H, J, K). In old leaves, this was the case for Zn and Mn under +Fe and −Fe (Fig. 4D, G) and for Cu under −Fe (Fig. 4J). In young leaves, it was the case for all elements, but in an Fe-dependent manner, such as for Fe and Mn under +Fe (Fig. 4B, H), and for Zn and Cu under both Fe supply conditions (Fig. 4E, K). We then compared the elemental contents at each stage between +Fe and −Fe in leaves. Surprisingly, there were not many differences in the old leaves when we compared +Fe and −Fe conditions across the stages. In old leaves, there were no differences in Fe, Zn, and Mn contents between +Fe and −Fe treatments and across the growth stages (Fig. 4A, D, G). However, there was a difference in Cu contents, which had increased at RS in the old leaves (Fig. 4J). In young leaves, on the other hand, the Fe contents had clearly decreased between +Fe and −Fe from RS to FS (Fig. 4B). The Cu contents had again increased between +Fe and −Fe at the RS (Fig. 4K). When comparing old and young leaves, the metal contents were either unchanged or lower in young versus old leaves in the cases of Fe, Zn, and Mn (Supplementary Fig. S6A–F). Only Cu amounts were higher in young than in old leaves at the BS under +Fe condition (Supplementary Fig. S6G). Hence, unlike young leaves, it seems that Fe was probably not mobilized from old leaves during the reproductive stages under −Fe, and Fe was also not likely to have moved from old to young leaves. This was different for Mn and Zn, which were probably mobilized in old and young leaves as their contents decreased with progression of reproduction.

The root elemental contents supported that root uptake was dependent on Fe supply. As expected, Fe contents were lower in roots under −Fe than +Fe, even though this only occurred at BS and FS (Fig. 4C). Unexpectedly, the content of Zn was lower at RS and that of Mn was lower at MS when comparing −Fe against +Fe (Fig. 4F, I). Notably, the Cu content was increased in −Fe versus +Fe roots at all stages (Fig. 4L), which presumably accounts for the elevated Cu contents in −Fe leaves at RS.

Hence, complex interactions in metal ion physiology were observed in old and young leaves under +Fe and −Fe across the stages. −Fe affected Fe, Zn, and Mn contents similarly, but had very different effects on Cu contents in both leaves and roots. The highest metal ion contents were mostly observed at the early reproductive stages, indicating a usage during progression to late reproductive development.

### Gene expression correlated with the mineral contents in leaves

**Fig. 5.**
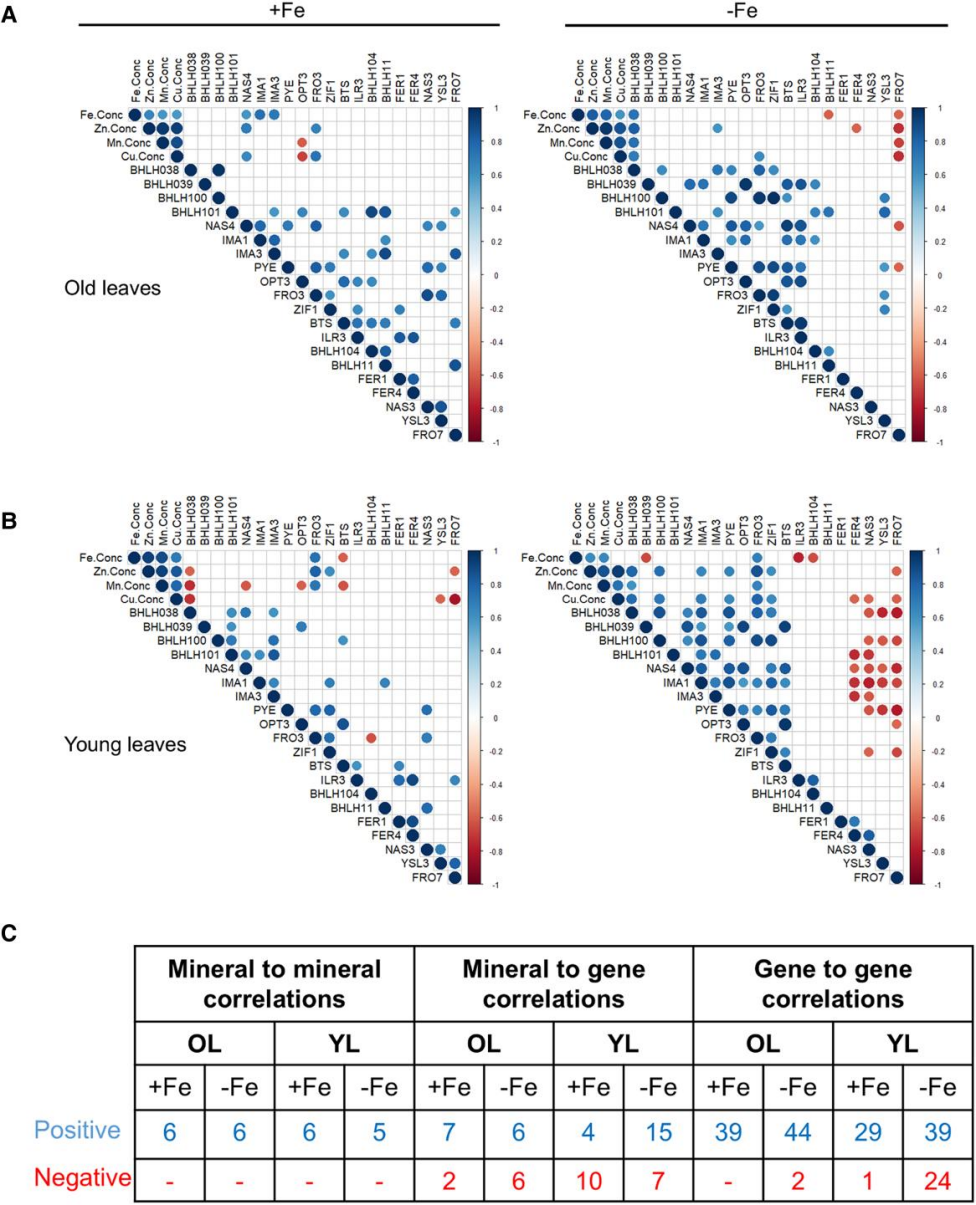
Correlation analysis between gene expression changes and ionomic contents. Correlations were performed with data from old leaves (A), and young leaves (B) under +Fe (right side) and −Fe (left side) conditions. Analysis was done using averages of normalized gene expression values and mineral concentrations of Fe (Fe Conc.), Zn (Zn Conc.), Mn (Mn Conc.), and Cu (Cu Conc.) across all four stages RS, BS, FS, and MS. The color scale represents Pearson’s correlation coefficients normalized in the range between −1 and 1, where −1 is negative correlations and 1 is positive correlations calculated between genes and mineral content pairs. Significant correlations at *P* < 0.05 are displayed in the diagram. (C) Summary table showing the number of correlations between either mineral-to-mineral content, mineral content to gene expression level, or correlation of gene-to-gene expression level in old and young leaves. Numbers show the total number of positive and negative correlations under both +Fe and −Fe conditions.

We investigated whether the gene expression levels correlated with mineral contents. Notably, we observed positive correlations between Fe, Zn, Mn, and Cu levels in both old and young leaves under +Fe and −Fe conditions. This confirmed an interaction in the mineral metal physiology in both leaf types under changing Fe environments. Furthermore, we found meaningful correlations between mineral metal contents and gene expression levels in old and young leaves (Fig. 5; Supplementary Fig. S7).

In old leaves, Fe showed the most significant correlations with genes under +Fe while Zn was the most correlated in comparison with other minerals under −Fe (Supplementary Fig. S7A, B). Fe contents correlated positively with the three co-expressed RED –Fe genes *NAS4*, *IMA1*, and *IMA3* under +Fe, and with *BHLH038* under −Fe (Fig. 5A; Supplementary Fig. S7A, B). Negative correlations were found under −Fe between Fe content and *BHLH11* and *FRO7* expression. Furthermore, in old leaves, Zn and Cu content correlated positively with expression of RED –Fe genes *NAS4* and *FRO3* under +Fe, and *BHLH038* under −Fe (Supplementary Fig. S7A, B). On the other hand, *OPT3* negatively correlated with Mn and Cu levels under +Fe conditions. Positive correlations were seen between Zn, Mn, and Cu amounts with *BHLH038* under −Fe, and negative correlations of the content of these minerals with *FRO7* expression (Fig. 5A; Supplementary Fig. S7B).

In young leaves (Fig. 5B), different correlations were seen, and most of these correlations were under −Fe. Interestingly Mn, Zn, and Cu were most correlated with genes under +Fe whereby Cu showed most correlations under −Fe conditions (Supplementary Fig. S7C, D). Fe contents correlated positively with *FRO3*, and negatively with *BTS* under +Fe (Fig. 5B; Supplementary Fig. S7C). At −Fe, the Fe amounts positively correlated with *FRO*3, and negatively with *BHLH039, ILR3*, and *BHLH104* gene expression (Fig. 5B; Supplementary Fig. S7D). There were positive correlations between Zn and Mn content and *FRO3* under +Fe. Under similar Fe conditions, negative correlations appeared of Zn and Cu with *BHLH038* and *FRO7*, while Mn negatively correlated with *BHLH038*, *NAS4*, *OPT3*, and *BTS* expression levels. When Fe was absent, more positive correlations were observed between Zn content with *BHLH038/100*, *IMA1*, *PYE*, *FRO3*, and *ZIF1*, but a negative correlation with *FRO7* (Fig. 5B; Supplementary Fig. S7D). Similar correlation patterns were observed with Cu amounts in the young leaves under −Fe. Additionally, Cu amounts showed negative correlations with *FER4*, *NAS3*, and *FRO7*. Similar to the old leaves, the contents of Fe, Zn, Mn, and Cu in young leaves positively correlated with each other under both Fe conditions, except between Fe and Cu under −Fe. A quantification of correlation between gene expression and mineral contents is shown in Fig. 5C.

In summary, the observed correlation patterns confirmed that the gene expression was indeed linked with the mineral metal contents in the old and young leaves under +Fe and −Fe conditions. It was also possible to distinguish old and young leaf Fe physiology by the number of correlations among gene expression and metal contents. The mineral metal levels correlated with each other and may account for the observed gene expression changes in the leaves.

## Discussion

In this study, we investigated how reproductive transitions impact Fe and metal ion physiology in rosette leaves when plants were exposed to +Fe and −Fe. Leaf growth, especially between RS and FS, was markedly influenced by Fe availability, which indicates an interesting cross-connection with leaf signaling events influencing leaf growth. Contrary to our idea that −Fe would cause important mobilization of Fe from rosette leaves during late reproductive stages, our findings revealed unexpected stage-specific dynamics in Fe and metal ion homeostasis. At the early reproductive stages, plants demonstrated a stronger response to −Fe in the rosette leaves than at late reproductive stages, suggesting that rosette leaves at late reproductive stages do not play important roles in metal ion homeostasis. Old and young leaves reacted differently in terms of changes of metal contents and gene expression patterns in response to +Fe and −Fe. The data indicate that old and young leaves can be sources and sinks for metal ions under different Fe conditions.

### Growth of young leaves is rapidly inhibited under –Fe, suggesting an intersection of –Fe and leaf growth signaling

In comparison to old leaves, young leaf expansion was notably slowed following a 3 d −Fe treatment, particularly during the rapid growth at the BS and FS (Fig. 1E). The inhibitory effect of −Fe on growth and greening of young leaves was very rapid and became visible already after 1 d of −Fe (Fig. 2B, D; Supplementary Fig. S2F). This fast Fe-dependent leaf growth response demonstrates the high plasticity of young leaves in response to environmental cues, in this case −Fe signals. Old leaves, instead, had already reached their final size so that their growth response was unaffected. Leaf growth is the result of cell proliferation and cell expansion (Wu *et al*., 2009; Xu *et al*., 2016; Li *et al*., 2024). We therefore believe that the observed reduced leaf areas of young leaves could be a consequence of repression of growth and plasticity in these leaves upon −Fe signals. Future studies will focus on dissecting the players at the intersection between regulation of leaf growth and Fe homeostasis control.

It is known that leaf form and morphology change in response to environmental and developmental cues, and are especially common for the vegetative phase change, which is caused by a process referred to as leaf heteroblastic reprogramming (Tsukaya *et al*., 2000; Tsukaya, 2005; Bäurle and Dean, 2006; Costa *et al*., 2012). In *A. thaliana*, SQUAMOSA PROMOTER BINDING PROTEIN-LIKE 9 (SPL9) targeted by miR156 regulates the juvenile-to-adult transition which influences leaf morphology (Wu *et al*., 2009; Xu *et al*., 2016). Mechanisms for leaf development transition and Fe homeostasis have been proposed for the miR156–SPL9 module, whereby SPL9 could transcriptionally regulate Fe deficiency markers *FIT* and *PYE* by direct promoter binding (Wang, 2015), a model requiring further investigation. Furthermore, in rice, *OsSPL9*, an ortholog of *SPL9* in Arabidopsis, has been proposed to be involved in Cu deficiency response whereby a knockout *spl9* mutant was sensitive to Cu deficiency with reduced accumulation of Cu in the old versus young leaves (Wang *et al*., 2024). These studies confirm that SPL9, among other leaf growth regulators, may indeed influence mineral uptake responses to synchronize development with nutrient availability. Thus, it is possible that in our system, −Fe signals influenced expression levels of leaf growth regulators. Future studies may investigate Fe responses using mutants defective in leaf cell proliferation and elongation, for example using plants with *SPL9* mutations which were shown to have defects in leaf cell expansion during the juvenile-to-adult transitioning (Li *et al*., 2024).

On the other hand, retrograde chloroplast signaling can also affect cell expansion and leaf greening (Andriankaja *et al*., 2012). Inferring from this, it is also a possibility that in our system, −Fe signals acted to inhibit leaf cell expansion and greening by blocking retrograde signaling. In such a scenario, −Fe signaling to the chloroplasts represses signals towards the nucleus and inhibits expression of nuclear-encoded leaf development- and photosynthesis-related genes. Previous studies have addressed retrograde signaling upon −Fe (Balparda *et al*., 2020, 2021). Alternatively, retrograde chloroplast signals may be activated under −Fe but then block the reprogramming in the nucleus, thus influencing downstream transcription of photosynthesis and growth regulators (Nam *et al*., 2021). Transcription factors induced by −Fe, including the class 1b bHLH proteins, might be able to interact with the transcriptional regulation apparatus for leaf cell differentiation, thus regulating leaf cell proliferation and expansion as demonstrated before (Andriankaja *et al*., 2014). TCP transcription factors, which positively control leaf expansion, can bind with −Fe-induced bHLH subgroup Ib proteins and transcriptionally repress their expression (Andriankaja *et al*., 2012). However, in these studies, bHLH subgroup Ib transcription factors seemed to be induced during the transition phase from cell proliferation to cell expansion under +Fe in early leaf growth phases. This appears contradictory to our −Fe data where these genes were also induced in old leaves (Fig. 3C). Nevertheless, it needs to be taken into account that these previous studies investigated seedling stages while we focused on later reproductive stages, which may account for such differences.

### Distinct Fe physiology in early and late reproductive stages and in old and young leaves

Rosette leaves display more distinctive Fe physiology responses under +Fe at the RS and BS than at late reproductive stages. The gene expression patterns and metal contents indicate that at the early reproductive stages, metal ion physiology is more active than at late reproductive stages. For example, Fe recycling genes (RED −Fe genes) *NAS4*, *OPT3*, and *FRO3*, which play a key role in Fe redistribution between source and sinks (Stacey *et al*., 2008; Schuler *et al*., 2012; Kumar *et al*., 2017; Khan *et al*., 2018; Nguyen *et al*., 2022), were up-regulated in both old and young leaves at RS and BS (Fig. 3C). This showed that Fe mobilization responses in old and young leaves were highly induced during these stages. Studies have demonstrated that Fe fluxes increase between the rosette and the inflorescence organs including stems, fruits, and seeds after onset of flowering (Waters and Grusak, 2008; Mendoza-Cózatl *et al*., 2014; Zhai *et al*., 2014; Pottier *et al*., 2019). On the other hand, high Fe genes, which are up-regulated by Fe sufficiency and excess conditions and down-regulated by Fe deficiency (Briat *et al*., 2010; Roschzttardtz *et al*., 2013), such as *FER1* and *NAS3*, were expressed similarly throughout the four stages in old and young leaves, respectively, while a marker for internal Fe mobilization from the chloroplast, *FRO7*, was predominantly expressed at the FS and MS in both leaves (Fig. 3B, C). This can indicate that Fe recycling from subcellular storage involving the RED −Fe genes is more important at the early reproductive stages. At these stages, old leaves can be sources of Fe and other metal ions for supply to sink organs, including developing leaves and inflorescence stems. At the later reproductive stages, Fe remobilization from plastids involving genes such as *FRO7* may be switched on. Fe is mobilized from plastids at the onset of leaf senescence and may prepare for Fe allocation towards seed filling (Himelblau and Amasino, 2001). Senescence takes place in the older leaves and can be triggered by plant age and environmental cues in order to enhance plant resilience to stress conditions and efficiently use nutrients (Nooden *et al*., 1996; Buchanan-Wollaston, 1997; Lemaître *et al*., 2008; Waters *et al*., 2009; Sperotto *et al*., 2012). Leaf senescence can lower the sensitivity to external −Fe as plants instead mobilize intracellular Fe stores in the vegetative organs instead of utilizing *de novo* uptake by the roots (Pottier *et al*., 2019). Additionally, the resulting oxidative stress responses due to internal Fe release may induce expression of the high Fe gene cluster in leaves, consequently dampening Fe uptake responses in roots (Le *et al*., 2016).

Rosette leaves are the targets of xylem mineral transport. There was a drastic decrease of Zn and Mn in old and young leaves and a decrease of Fe and Cu contents in young leaves under +Fe from RS towards MS (Fig. 4A–K), indicating that at late reproductive stages metals may have been exported from the rosette leaves to replenish sinks forming in the inflorescence such as fruits and seeds. It is likely that at the late reproductive stages the xylem stream is directly oriented towards the inflorescence stem, rather than the rosette leaves, so that cauline leaves and inflorescence stems account primarily for allocation of Fe taken up *de novo* and transported from roots to seeds (Waters and Grusak, 2008; Schuler *et al*., 2012; Pottier *et al*., 2019). The enhanced −Fe responsiveness in the shoot apex samples even at FS and MS speaks in favor of this. In future studies it can be investigated whether new Fe storage tissues arise during reproductive growth in addition to the rosette leaves, for example in the secondary phloem tissues, coupled with secondary growth in the Arabidopsis hypocotyl during flowering (Lehmann and Hardtke, 2016; Wunderling *et al*., 2018).

Notably, particular observations were highly interesting and striking. One remarkable observation was that the differential expression of RED −Fe genes in the old leaves under −Fe indicated a subfunctionalization among RED −Fe gene functions (Fig. 3C). The expression regulation of subgroup Ib *BHLH*, *IMAs*, and *NAS4* genes could be distinguished from that of *PYE*, *OPT3*, *FRO3*, *ZIF1*, and *BTS*. PYE negatively regulates certain RED −Fe genes, including *bHLH1B* genes, *FRO3*, *ZIF1*, and *NAS4* in seedlings (Long *et al*., 2010; Haydon *et al*., 2012; Schuler *et al*., 2012; Pu and Liang, 2023). Possibly, individual regulator proteins such as PYE, BTS, or upstream transcription factors have individual functions in controlling positively or negatively different subsets of Fe mobilization and Fe storage genes in different tissues (Zhang *et al*., 2015; Rodríguez-Celma *et al*., 2019; Akmakjian *et al*., 2021; Muhammad *et al*., 2022) and at different developmental stages. Future studies can focus on investigating the roles of regulatory genes not only during early vegetative seedling stages but also during reproductive stages.

The expression of BLUE –Fe genes in roots was induced in the old leaves, young leaves, and shoot apex from the RS to MS under −Fe conditions (Supplementary Fig. S5B). This shows age-dependent up-regulation, especially with progression from early to late reproductive development. Indeed, reports have shown evidence of mRNA transport between shoots and roots; for example, *IRT1* was among the transcripts transported a long distance in grafted plants grown under nutrient limitation (Thieme *et al*., 2015). BLUE –Fe genes can be expressed in shoots, even though this has not yet been explored. It has been shown that *IRT1* and *FRO2* are expressed in seedling leaves, even though to a lesser extent compared with roots (Jakoby *et al*., 2004). Moreover, *IRT1* promoter-driven reporter activity was observed in flowers (Vert *et al*., 2002). Furthermore, overexpression of FIT led to ectopic expression of *IRT1* and *FRO2* in leaves under +Fe and −Fe conditions (Meiser *et al*., 2011). This was also demonstrated in BHLH39Ox plants whereby *FIT* expression was evident in leaves of seedlings, and surprisingly to a similar level to that in wild-type plants (Naranjo-Arcos *et al*., 2017). This indicates that FIT and its direct target genes, *IRT1* and *FRO2*, are expressed in shoots, too. Thus, there is a possibility that BLUE −Fe genes are induced in leaves and reproductive meristems during later reproductive stages. Future studies should further explore the role of this age-dependent expression pattern.

Another observation was interesting, namely the increase of Cu contents in old and young leaves at the RS following the −Fe treatment (Fig. 4J, K). This Cu increase was certainly linked with the very high Cu uptake into roots under −Fe (Fig. 4L). It indicates that despite their growth inhibition, the young leaves accumulated high Cu amounts at this stage. There is evidence for Fe and Cu crosstalk whereby Cu amounts in roots and shoots are increased under −Fe, and vice versa (Bernal *et al*., 2012; Carrió-Seguí *et al*., 2019; Chia *et al*., 2023; Waters and Armbrust, 2013; Kastoori Ramamurthy *et al*., 2018; Rai *et al*., 2021). One of the genes encoding a transporter for Cu uptake is *COPT2* which is a FIT target and up-regulated under −Fe (Colangelo and Guerinot, 2004). FIT protein also interferes with Cu regulators (Perea-García *et al*., 2020; Cai *et al*., 2021) . This observation raises the question of whether the gene expression changes and leaf growth suppression can truly be attributed to −Fe or whether they could be a secondary consequence of elevated Cu. Nicotianamine is a metal chelator for Cu and Fe, and Cu homeostasis is equally important as Fe for reproduction (Sheng *et al*., 2021). −Fe was also reported to cause Zn and Mn accumulation in seedlings due to the elevated IRT1 activity leading to the uptake of other bivalent metals including Fe, Zn, and Mn (Dubeaux *et al*., 2018; Cointry and Vert, 2019). Since we did not see this effect of Zn and Mn accumulation in −Fe in roots and rosette leaves at the reproductive stage, except for elevated Mn at MS in roots, it is possible that IRT1 function has a different relevance during reproduction or that mechanisms are in place to repress metal uptake and transport to rosette leaves targeting Mn and Zn, thus preventing overaccumulation.

## Future perspectives

The results of this study indicate a clear and significant negative impact of Fe deficiency on rosette and young leaf growth dynamics, as well as the mineral partitioning in *A. thaliana* during the reproductive stages. The findings underscore the crucial role of Fe in plant growth at reproductive stages and complex interactions and signaling between the various plant parts. This underlines that more attention should be paid to the roles of Fe homeostasis genes in the reproductive stages. Further analysis is thus required to elucidate the molecular mechanisms acting during reproduction. Our study was limited by two aspects. On the one hand, only wild-type Col-0 plants were investigated. Future studies will focus on natural variation and mutants of individual genes to undermine their roles in reproduction. On the other hand, the conclusions are based on gene expression and metal contents, excluding the physiological roles of the encoded transport proteins and enzymes. These components are subject to post-translational and protein control, and clearly mutants affected in such regulatory mechanisms can reveal the contributions during reproductive stages.

The question arises of whether old leaves can be considered as Fe sources and whether young leaves are Fe sinks under −Fe (Hantzis *et al*., 2018; Nguyen *et al*., 2022; Swartz *et al*., 2023). The growth system established here can be utilized to test such questions by performing Fe supply experiments in combination with mutants that cannot steer Fe physiology properly at the reproductive stage. In the future, it will be interesting to investigate the molecular mechanisms potentially regulating systemic Fe signals from source to sinks in the rosette, and link gene expression with leaf growth signaling under +Fe and −Fe, for example by utilizing *SPL9* and /or *miRNA156* mutants or investigating the role of retrograde signaling in leaf-to-leaf communication.

Finally, analyzing gene expression at the reproductive phase is challenging, since variations in gene expression are relatively high and the whole-plant physiology is far more complex than in young seedlings. Whole-transcriptome studies will offer better insights into the general physiology of old and young leaves and more complete investigation of Fe homeostasis genes in the future.

## Supplementary Material

eraf207_Supplementary_Data

## Data Availability

The primary data supporting this study were not made publicly available at the time of publication. The data that support the findings of this study are available from the corresponding author upon request.

## Supplementary data

The following supplementary data are available at *JXB*  online.

Fig. S1. Materials collected from Arabidopsis Col-0, showing additional plant phenotypes at various plant growth stages.

Fig. S2. Additional radial charts showing individual leaf sizes at RS after different time point exposure to +Fe and −Fe treatment.

Fig. S3. Principal component analysis (PCA) with separation of old leaves, young leaves, shoot apex, and roots according to gene expression under +Fe (control) or −Fe conditions at RS, BS, FS, and MS.

Fig. S4. Hierarchical clustering of the expression pattern of selected 23 Fe deficiency-responsive genes in roots, old leaves, young leaves, and inflorescence shoot apex under +Fe (control) and −Fe.

Fig. S5. RT–qPCR analysis of Fe-regulated genes across the four stages in roots and shoots, displayed in heatmaps.

Fig. S6. Comparison of mineral contents in old (OL) and young (YL) leaves under +Fe and −Fe growth conditions.

Fig. S7. Network analysis of correlations between genes and minerals Fe, Mn, Zn, and Cu in old and young leaves under +Fe and −Fe conditions.

Table S1. List of primers used for gene expression analysis by RT–qPCR.
