# Supplementary material for: Age-dependent differential iron deficiency responses of rosette leaves during reproductive stages in Arabidopsis thaliana
Source: J Exp Bot. 2025 May 21;76(16):4598–614. doi: 10.1093/jxb/eraf207 (PMC12509885; doi:10.1093/jxb/eraf207)
Supplement: eraf207_Supplementary_Data [file eraf207_supplementary_data.zip › jexbot314556-file001.pdf]

Table S1. Primers for qPCR.

Gene name	AGI	RT primer pair (5'-3')	STD primer pair (5'-3')
EF1B-α2 (Reference gene)	AT5G19510	TATGGGATCAAGAAACTCACAAT<br>CTGGATGTACTCGTTGTTAGGC	GCTGCTAAGAAGGACACCAAG<br>TGTTCTGTCCCTACTGGATCC
EF1B-α2 (Genomic DNA control)	AT5G19510	TCCGAACAATACCAGAACTAC<br>CCGGGACATATGGAGGTAAG	GCTGCTAAGAAGGACACCAAG<br>TGTTCTGTCCCTACTGGATCC
IRT1	AT4G19690	AAGCTTTGATCACGGTTGG<br>TTAGGTCCCATGAACTCCG	TAGCCATTGACTCCATGGC<br>AGAAAACTATGAATCGTGGGG
FIT	AT2G28160	CCCTGTTTCATAGACGAGAACC<br>ATCCTTCATACGCCCTCTCC	AAGACATGACCAAAAATGTGTGT<br>TGCATCTCCAACAATGGATGC
FRO2	AT1G01580	CTTGGTCATCTCCGTGAGC<br>AAGATGTTGGAGATGGACGG	CCATGCTCGATCTTGTCTTG<br>ATTCCGGAACTTTTGAAAGG
bHLH38	AT3G56970	AGCAGCAACCAAAGGCG<br>CCACTTGAAGATGCAAAGTGTAG	GGAGATAACCTAAATAACGGCA<br>GGTCCAGATCAGTGTTAGATTCA
bHLH39	AT3G56980	GACGGTTTCTCGAAGCTTG<br>GGTGGCTGCTTAACGTAACAT	AACCAAAGCAGCTTCCAAG<br>CGAAGAGAAAAAGGACGACA
bHLH100	AT2G41240	AAGTCAGAGGAAGGGGTTACA<br>GATGCATAGAGTAAAAGAGTCGCT	GCCTTGCGGAGATCATAGCT<br>TCGCGTTGTTCTCCTTCCAA
bHLH101	AT5G04150	CAGCTGAGAAACAAAGCAATG<br>CAGTCTCACTTTGCAATCTCC	CATCCCATCAAAGTCTCTCTAGC<br>CCTCCAGTCTCACTTTGCAAT
bHLH11	AT4G36060	TCCATGCCTCTTCTTTAGC<br>GGAACTATCTTGAACAGCTTG	GAGTGACAAAGCCTCAGTTC<br>CATCGGTCATAGACAATAGTTCAG
PYE	AT3G47640	GTTCCCAGGACTTCCCATTT<br>GTGTCTGGGGATCAGGTTGT	AACGCACCACCTTCTTCTGT<br>GTAGCCGAGAAGACCACGAG
bHLH104	AT4G14410	GTTGAGGAGGGAGAAGCTAAATG<br>ACGGATTGCATCATCGAGTATAGC	GAATTTGCAGCAGGAGCCAG<br>GCCAAACGGAAGAATCCTAAACCG
ILR3	AT5G54680	GCATGTAGAGAGAAGCAGCGAC<br>TGCGGACAGCATCAACCAAG	TGATGGCTCGGCTGGAAAC<br>CTAAGAAAGCCGAGAAAGAGAGGAG
YSL3	AT5G53550	CTTGGAATATGAGAGATCGAGTTAA<br>CGAATATTTACTCGGCATGAA	ATTGTATCGATTTCTTCTGACCTAAT<br>AATATGACGTGTGCTGAATTGAC
OPT3	AT4G16370	CCCAAACAAGAAGTGGATCCC<br>GTGACCAACCAGCTGGCAAT	TCGGTTATATCCTGCCTG<br>GACAGATGTCTCAATAGCTC
IMA1	AT1G47400	TGCTTCCACCGTGTATGTTG<br>CAGGAGCATAATCATAGCCACTG	GGCCATCAAGAGATTTGACC<br>TGGAAACCATGTTTGTTCATCT
IMA3	AT2G30766	GGCAGGCTATACGAATCAACTC<br>CGTCACAGTCATCGTCGTCA	ACGCAGAAGGCAGGCTATAC<br>ACAGGCACGATCTACAACTTCA
FRO3	AT1G23020	ATCGACCACCTTGCTGTTTC<br>TTATCCCACTGCCTCCACTC	AATCAGATCGACCACCTTGC<br>TTCTTTTGGTGAGAAGATTTTGG
BTS	AT3G18290	CGGGGAAGGACTAGGAATCG	AACTTGGATGTTCCCCGTCT

		CAGCAGATGGGGCAATTTGT	ATCAACGGGCTTCTTCACAT
ZIF1	AT5G13740	TTGGCTGAGAAACTGCTAGG	AAGGCTTCTCAGTCTCTCTTG
		CTTAGACTGAGACCTGACAAGC	TAACGGTTCAAGTAAGTTCCTCTC
FRO7	AT5G49740	TGGTTACAGTGGCATCCTTTCA	TCGCTGTTTTACCCGGAGTT
		GCTGGTCTTGATTTTCTGCCTC	TCCGTTGGTCCATGGTGTTT
NAS3	AT1G09240	CAATTGGGAATGTTGGTGG	GCATGTTCTTCCACACCGTT
		TGTTCCTCCCTAGCTCCG	CGTGTTCTGTTTCAGCCCAA
NAS4	AT1G56430	TGTAATCTCAAGGAAGCTAGGTG	CACTCTCTTCAAGCAGCTCGT
		GCGAACTCCTCGATAATGC	CTGTAGCAAAAACAGCCAACA
FER1	AT5G01600	TAAGCCACTACTCCCTCACG	GCGGCTCAACACTATCCTCT
		TTGTTTGTGTCCACCGTAGC	ACAGAGCCAACTCCATTGCT
FER4	AT2G40300	TCTCGTCCCTACCAGCTCTC	TGGCGTGAAGAAGGATGTGT
		CAAGACCTTTGAGCGCGATG	GCGTTTAAACCGGAGCAAACT

Table S1. List of primers used for gene expression analysis by reverse transcription-qPCR. Primers were designed as previously described in (Ngigi & Bauer, 2023). Mass standard (STD) and real time qPCR (RT) primers annealed to the indicated genes. Mass standard PCR products were used for absolute quantification of transcript abundances of the respective genes.
