# Supplementary material for: Age-dependent differential iron deficiency responses of rosette leaves during reproductive stages in Arabidopsis thaliana
Source: J Exp Bot. 2025 May 21;76(16):4598–614. doi: 10.1093/jxb/eraf207 (PMC12509885; doi:10.1093/jxb/eraf207)
Supplement: eraf207_Supplementary_Data [file eraf207_supplementary_data.zip › jexbot314556-file002.pdf]

Supplemental material

Age-dependent differential iron deficiency responses of rosette leaves during reproductive stages in *Arabidopsis thaliana*

Short title: Differential iron deficiency responses of rosette leaves during reproduction

Mary Ngigi, Mather Khan, Ricarda Remus, Shishir K Gupta, Petra Bauer

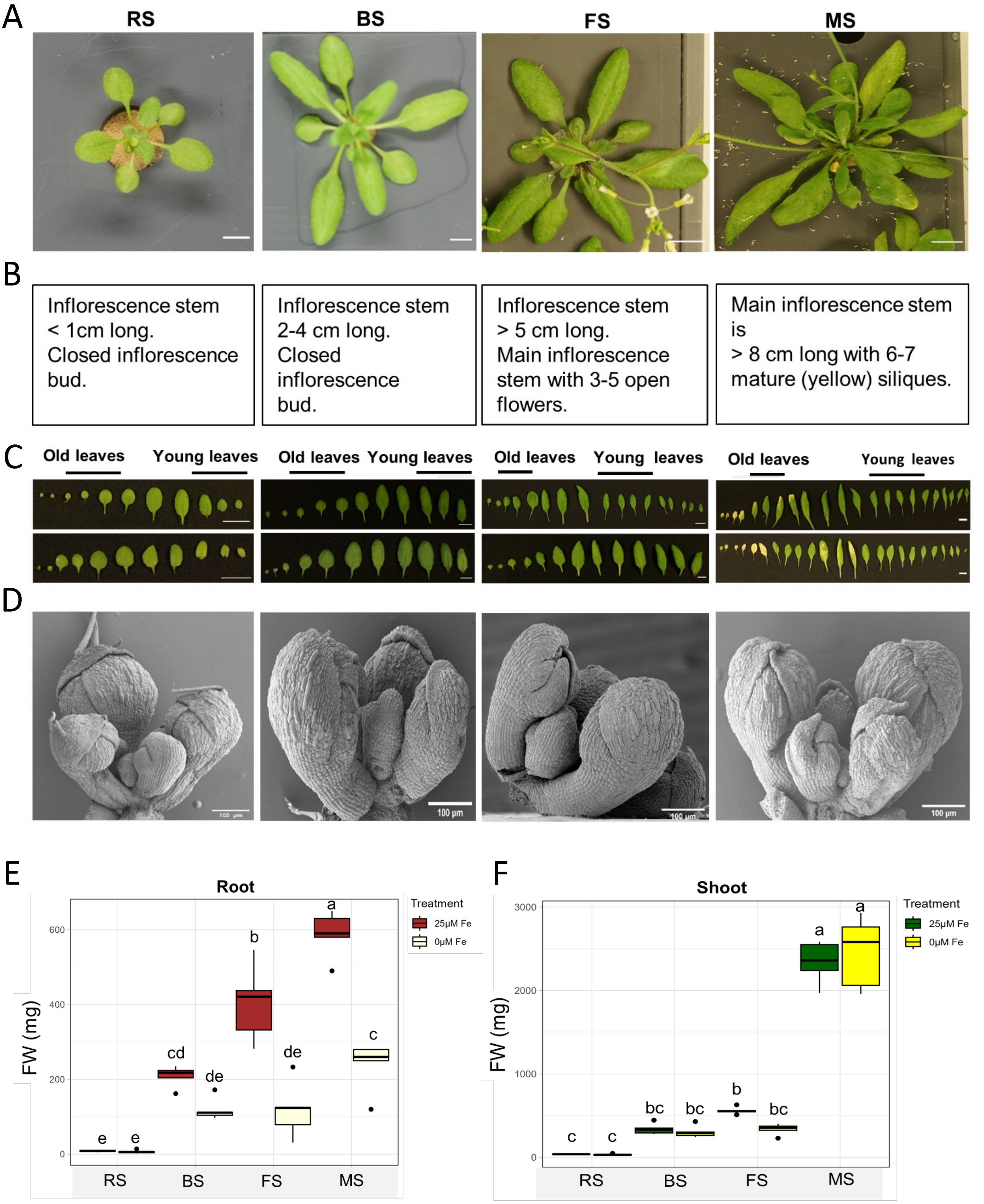
A
RS
BS
FS
MS
B
Inflorescence stem < 1cm long. Closed inflorescence bud.
Inflorescence stem 2-4 cm long. Closed inflorescence bud.
Inflorescence stem > 5 cm long. Main inflorescence stem with 3-5 open flowers.
Main inflorescence stem is > 8 cm long with 6-7 mature (yellow) siliques.
C
Old leaves
Young leaves
D
100 µm
E
Root
FW (mg)
0
200
400
600
RS
BS
FS
MS
Treatment
25µM Fe
0µM Fe
a
b
cd
de
c
e
F
Shoot
FW (mg)
0
1000
2000
3000
RS
BS
FS
MS
Treatment
25µM Fe
0µM Fe
a
b
bc
c

Figure S1. Materials collected from *Arabidopsis* Col-*0*. (A) Top view of plants with their rosette leaves at each stage, scale bar = 1cm. (B) Descriptions of the growth stages distinguishing phenotypes as described by **Boyes *et al.,* 2001**. (C) Old (left) and young (right) leaves collected from plants at the respective stages under +Fe (control) and –Fe after 3 days, scale bar = 1cm. (D) Inflorescence shoot apical meristem dissected from the main stem of plants at the rosette to mature stages., scale bar = 100 µm. (E-F) Root and shoot fresh weight of plants grown to RS, BS, FS, and MS and transferred to hydroponic media with +Fe or -Fe for 3 days. Data represents mean of three biological replicates (n = 15). Error bars show ± SD, one-way ANOVA and multiple comparison by Tukey test.

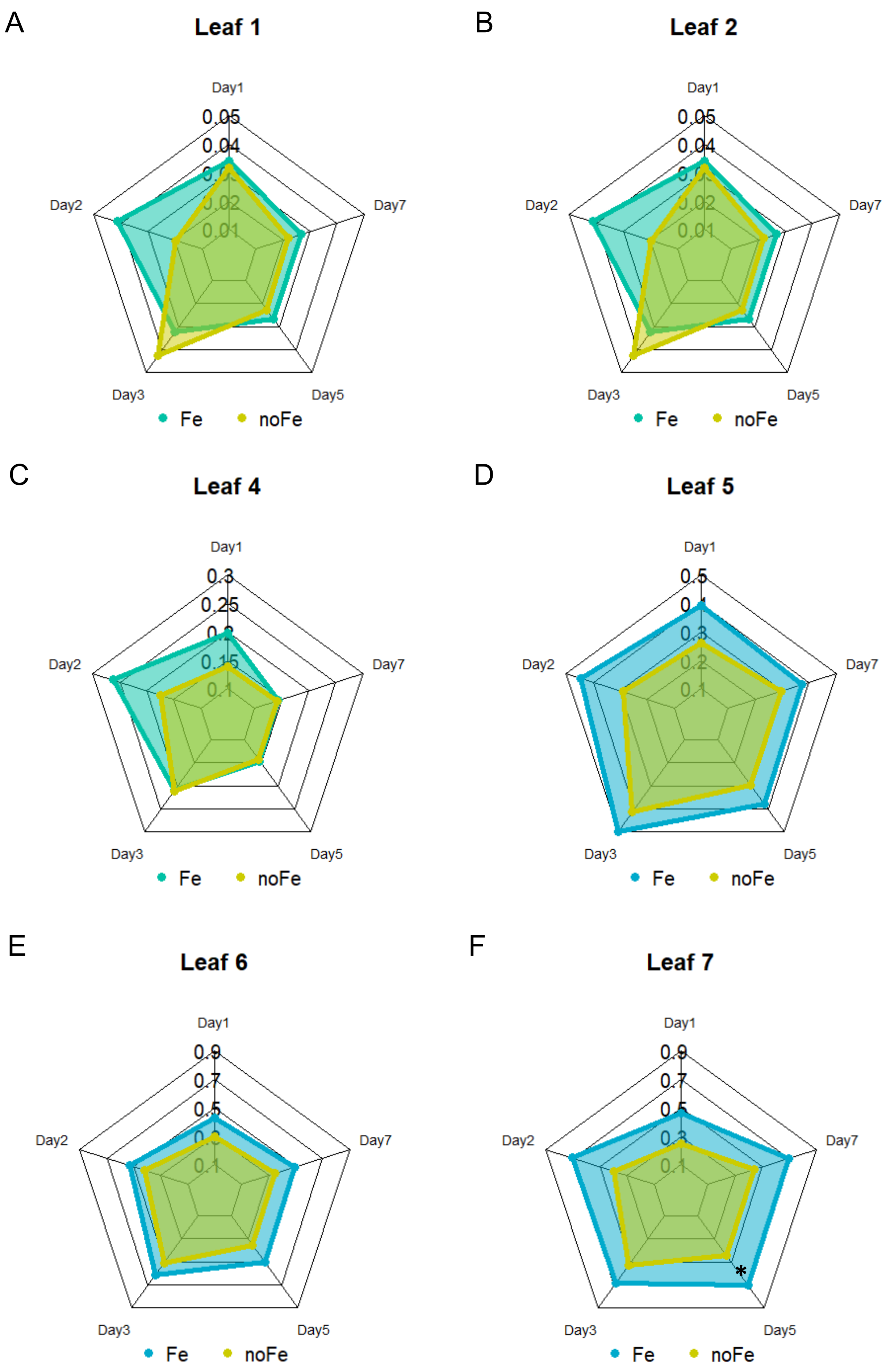
A
Leaf 1
Day1
Day2
Day3
Day5
Day7
0.05
0.04
0.02
0.01
Fe
noFe
B
Leaf 2
Day1
Day2
Day3
Day5
Day7
0.05
0.04
0.02
0.01
Fe
noFe
C
Leaf 4
Day1
Day2
Day3
Day5
Day7
0.3
0.25
0.15
0.1
Fe
noFe
D
Leaf 5
Day1
Day2
Day3
Day5
Day7
0.5
0.3
0.2
0.1
Fe
noFe
E
Leaf 6
Day1
Day2
Day3
Day5
Day7
0.9
0.7
0.5
0.1
Fe
noFe
F
Leaf 7
Day1
Day2
Day3
Day5
Day7
0.9
0.7
0.5
0.1
*
Fe
noFe

Figure S2. Individual sizes of leaves at RS after +Fe and -Fe. (A-F) Individual leaf areas of plants at RS after exposure to either Fe sufficient (+Fe) or deficient (-Fe) conditions from day 1 to day 7. Green and blue colors correspond to old and young leaf color scheme. Data represents average of biological replicates (*n*=3). Radial charts were plotted in R Studio using “fsmb” package. Asterisks show where leaf areas were statistically significantly different between +Fe and -Fe conditions, ($p<0.05$), one-way ANOVA and Tukey’s HSD test.

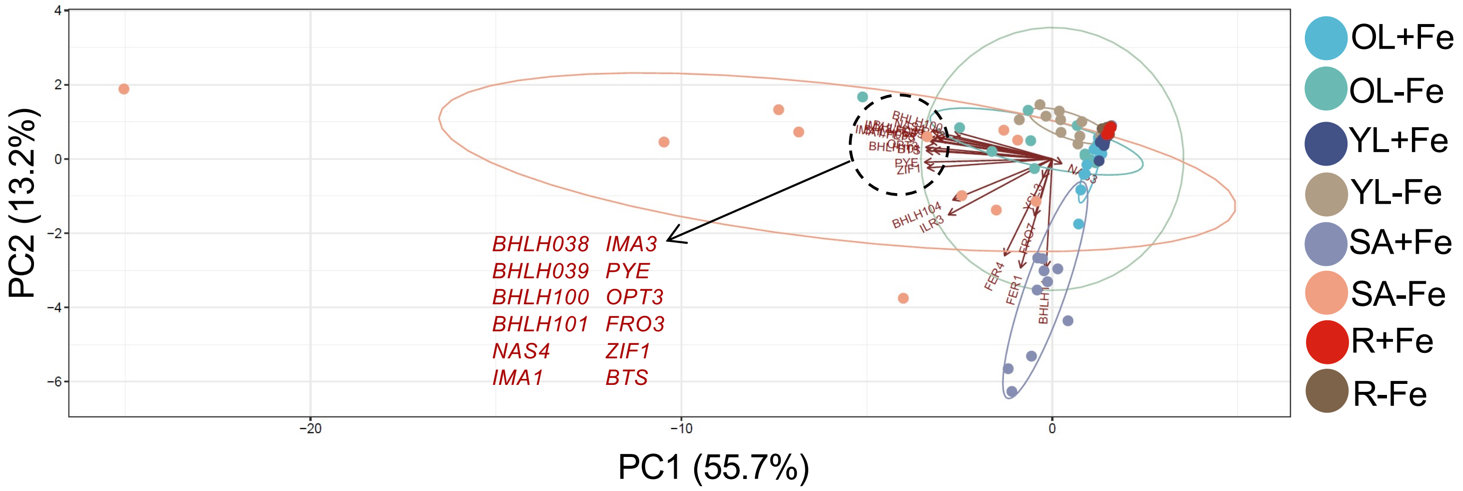

Figure S3. Principal component (PC) analysis with separation of old leaves, young leaves, shoot apex and roots according to gene expression at +Fe (control) or –Fe conditions at growth stages RS, BS, FS and MS. Samples are labeled as OL – old leaves, YL – young leaves, R – roots and SA – shoot apex. PC1 & PC2 separated samples according to growth stages and Fe condition. PC analayis was computed using normalized absolute expression values of Fe-response genes.

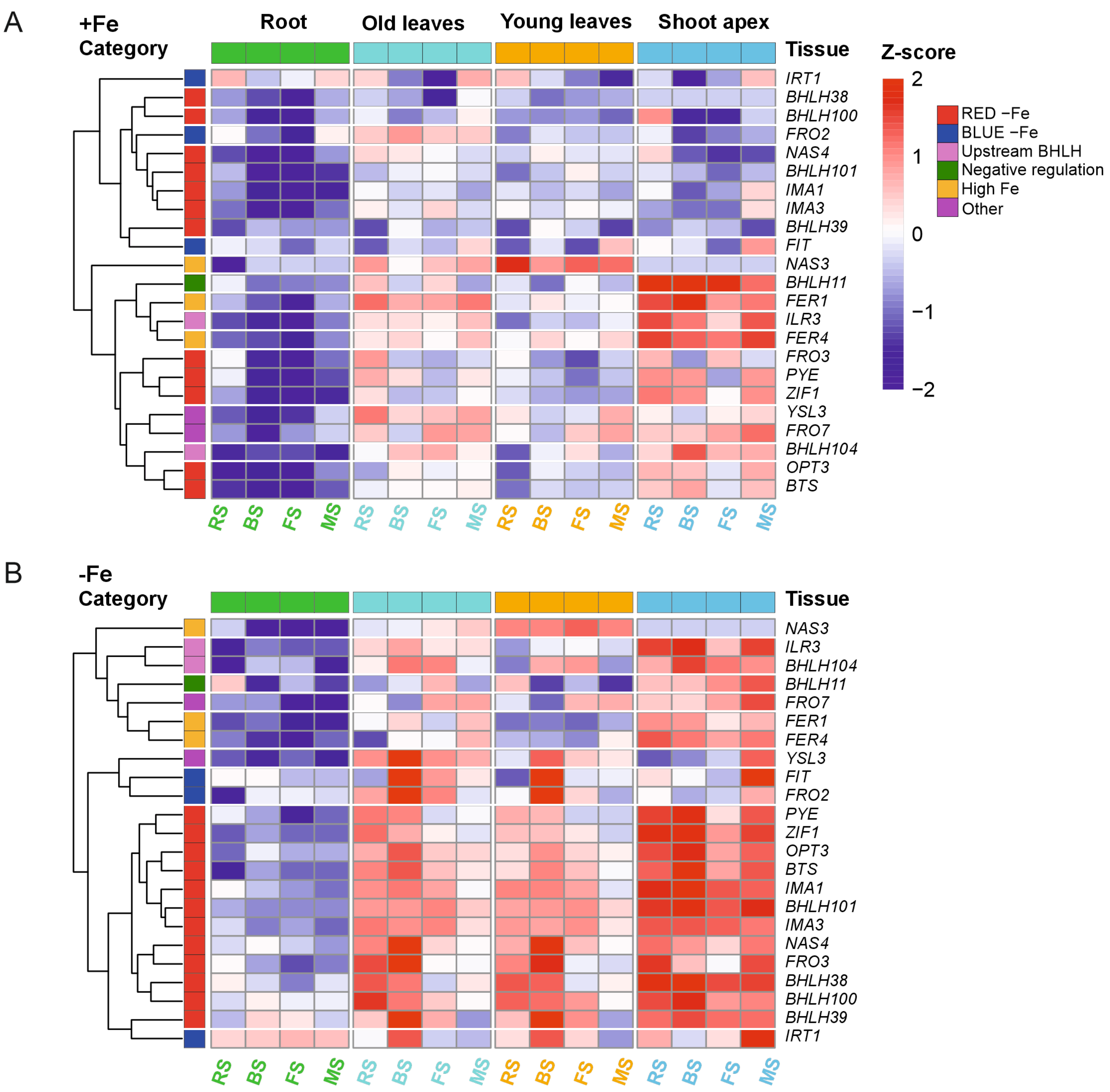

Figure S4. (A-B) Hierarchical clustering of the expression pattern of selected 23 Fe-deficiency responsive genes in roots, old leaves, young leaves, and inflorescence shoot apex at +Fe (control) and –Fe respectively, for 3 days during four stages; RS, BS, FS and MS. Gene category annotations are displayed on the left side and tissue-type annotations on the top. Gene upregulation is represented in red color and gene downregulation with blue colors, $p < 0.05$ ($n = 3$). The color scale ranges from -2 to 2 to represent the variation in gene expression.

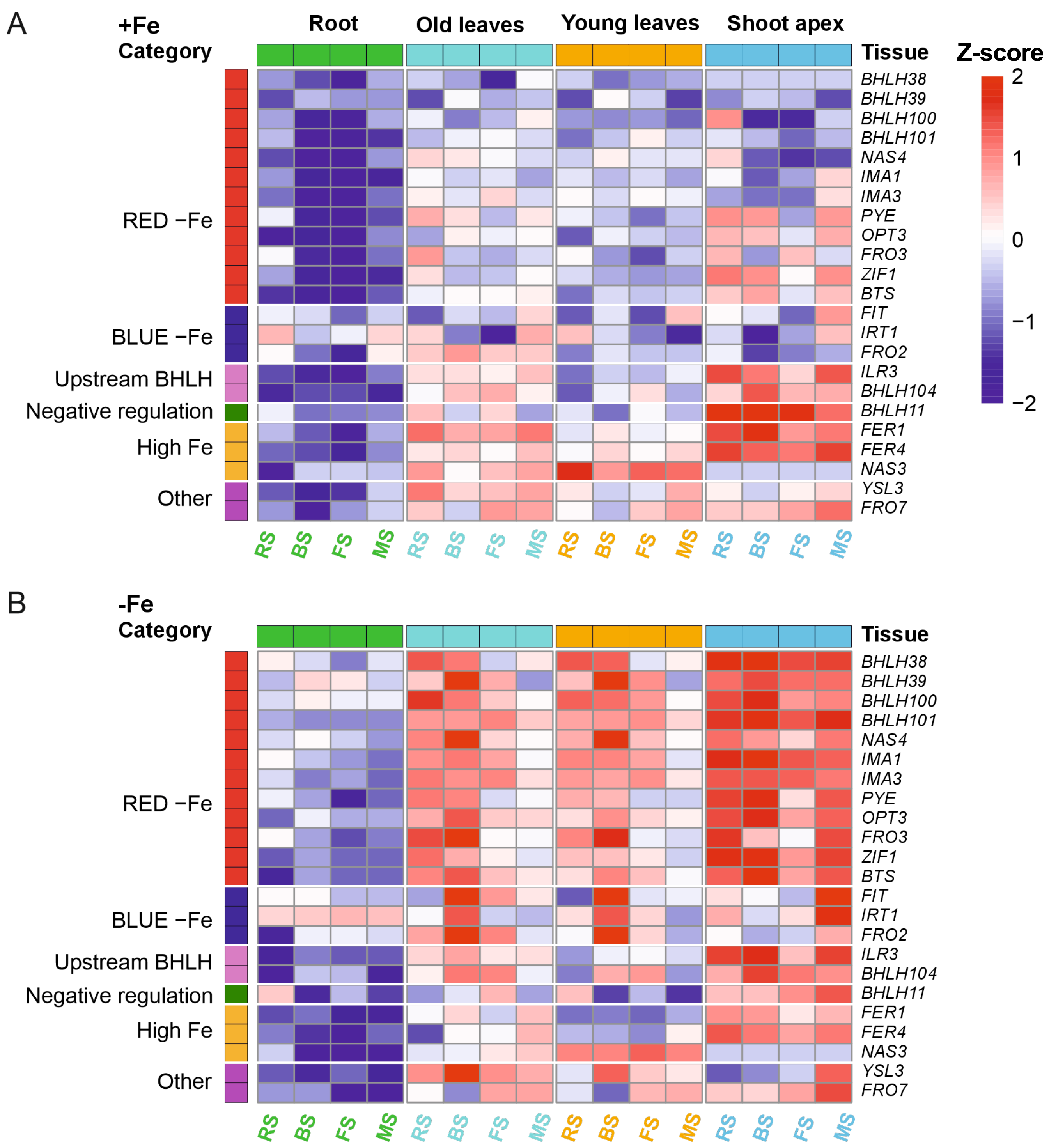

Figure S5. RT-qPCR analysis of Fe regulated genes across the stages. (A-B) Heatmaps showing the absolute expression profile of 23 Fe-response markers in roots, old leaves, young leaves, and inflorescence shoot apex at +Fe (control) and –Fe respectively, for 3 days during four stages; RS, BS, FS and MS. Functional gene category annotations are displayed on the left side of the heatmap, with tissue-type annotations displayed on the top. Gene upregulation is represented in red color and gene downregulation with blue colors, $p < 0.05$ ($n = 3$). The color scale ranges from -2 to 2 to represent the variation in gene expression.

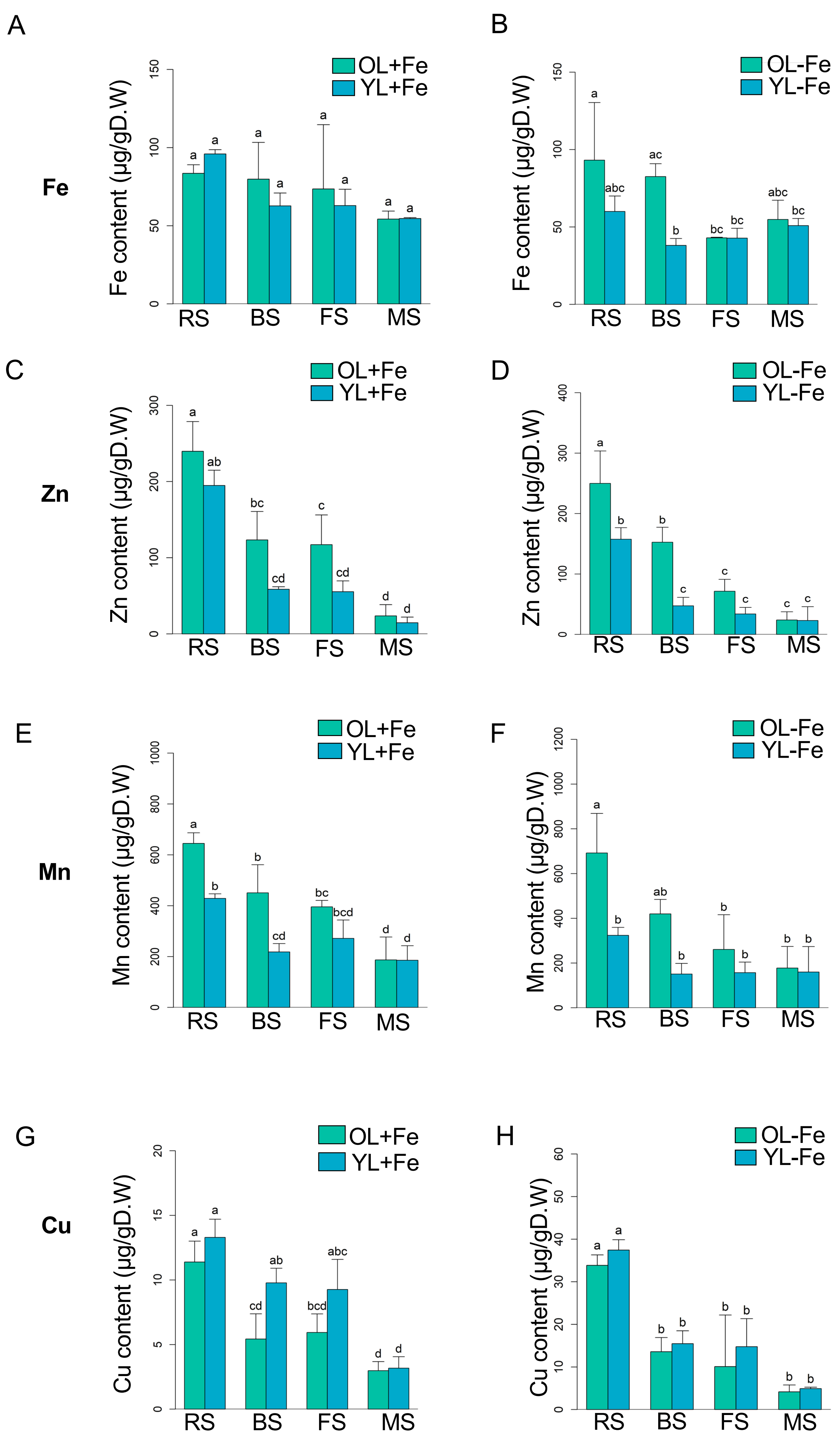

A
B
C
D
E
F
G
H
Fe
Zn
Mn
Cu
OL+Fe
YL+Fe
OL-Fe
YL-Fe
Fe content (μg/gD.W)
Zn content (μg/gD.W)
Mn content (μg/gD.W)
Cu content (μg/gD.W)
RS
BS
FS
MS

Figure S6. Comparison of mineral contents in old (OL) and young (YL) leaves at +Fe and –Fe growth conditions. Fe (A-B), Zn (C-D), Mn (E-F), and Cu (G-H) content in old leaves and young leaves at +Fe or –Fe growth conditions at the growth stages; RS, BS, FS and MS. Plants were exposed to 3 days of +Fe or –Fe before sample collection. Data represents mean of three biological replicates (*n* = 15). Error bars represent ± SD. Letters on top of each bar show level of significance according to one-way ANOVA and Tukey's HSD test, $p < 0.05$.

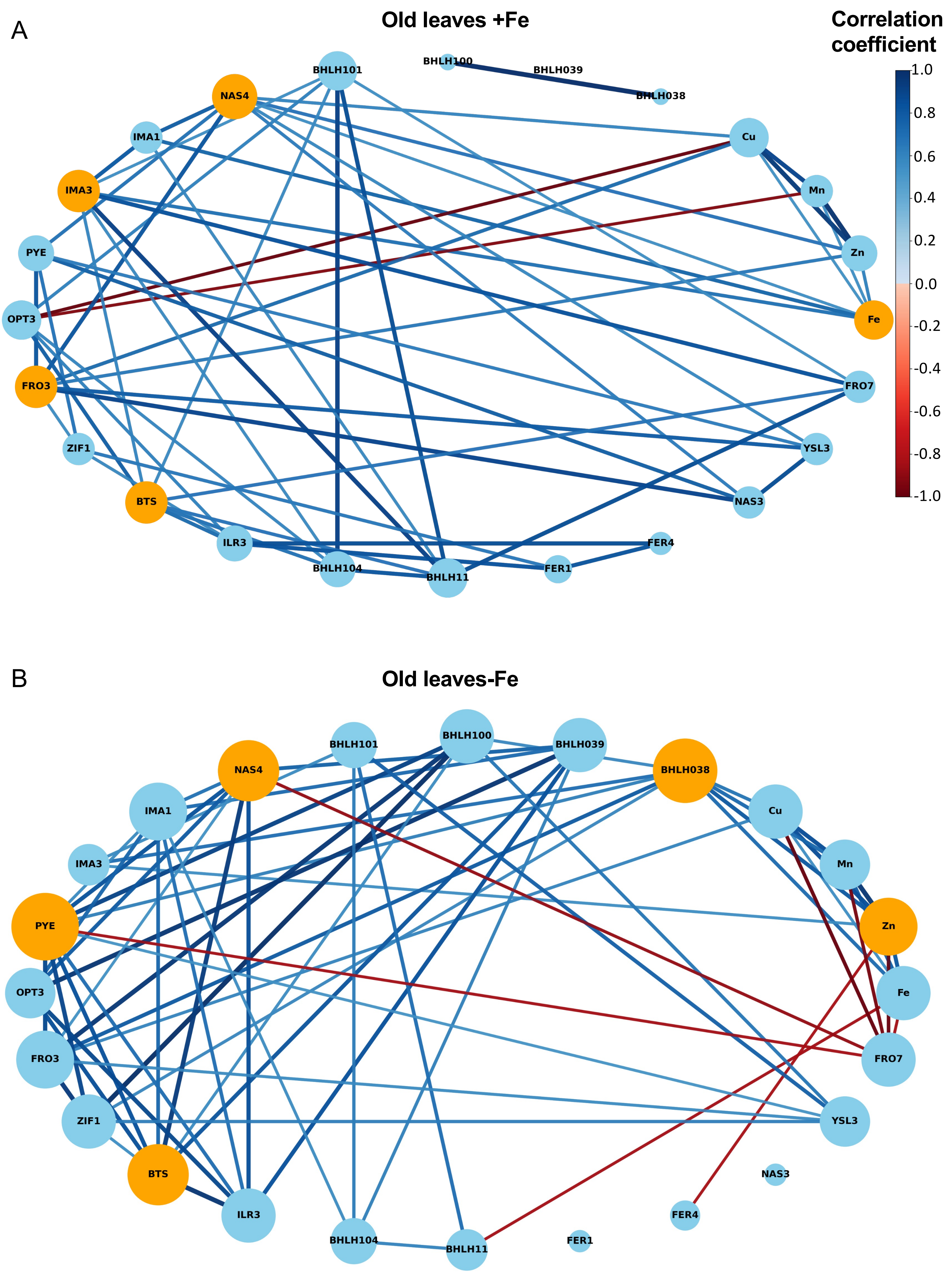
A
Old leaves +Fe
Correlation coefficient
1.0
0.8
0.6
0.4
0.2
0.0
-0.2
-0.4
-0.6
-0.8
-1.0
BHLH101
BHLH100
BHLH039
BHLH038
NAS4
IMA1
IMA3
PYE
OPT3
FRO3
ZIF1
BTS
ILR3
BHLH104
BHLH11
FER1
FER4
NAS3
YSL3
FRO7
Fe
Zn
Mn
Cu
B
Old leaves-Fe
BHLH101
BHLH100
BHLH039
BHLH038
NAS4
IMA1
IMA3
PYE
OPT3
FRO3
ZIF1
BTS
ILR3
BHLH104
BHLH11
FER1
FER4
NAS3
YSL3
FRO7
Fe
Zn
Mn
Cu

C

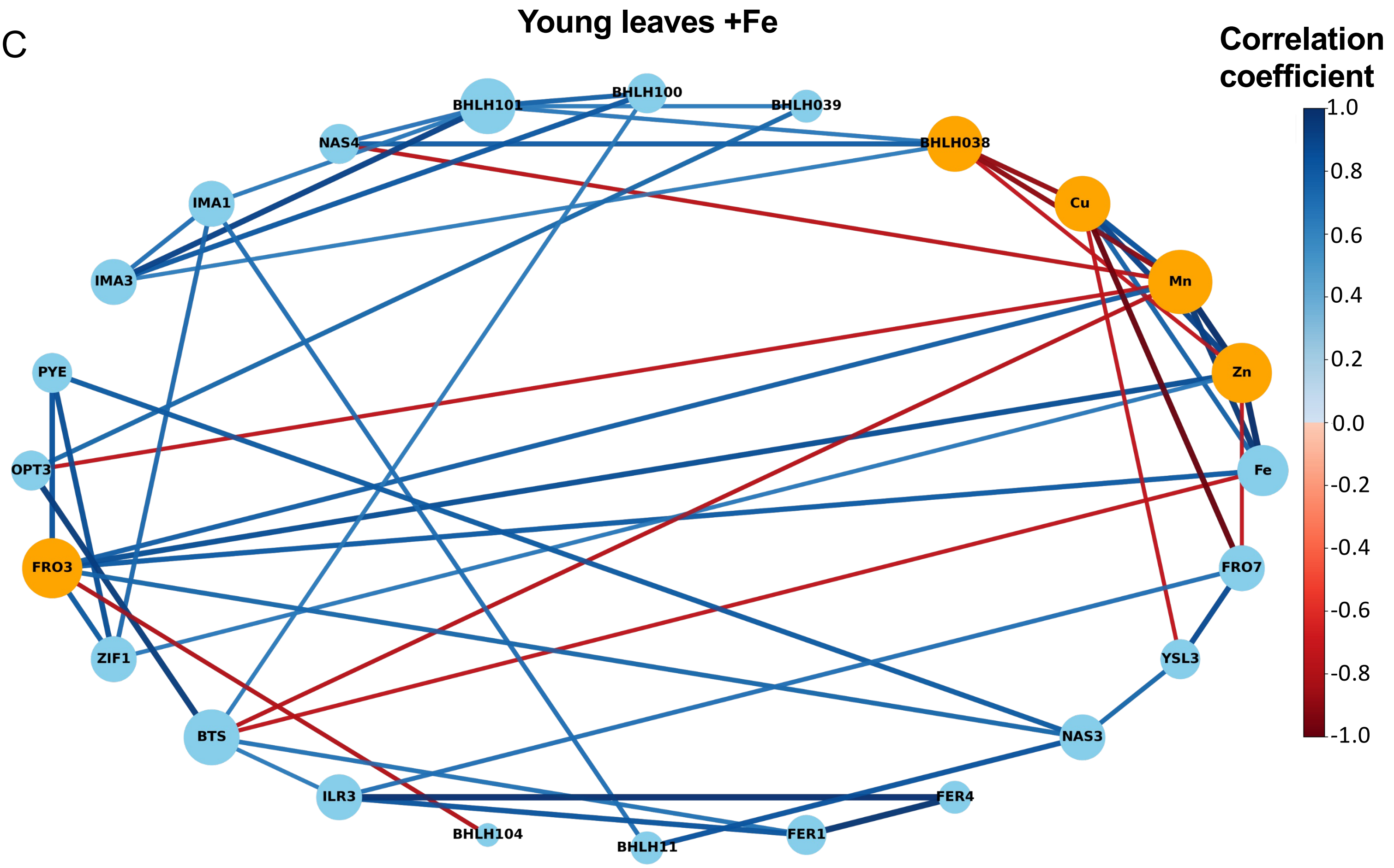
Young leaves +Fe
Correlation coefficient
1.0
0.8
0.6
0.4
0.2
0.0
-0.2
-0.4
-0.6
-0.8
-1.0
BHLH101
BHLH100
BHLH039
NAS4
BHLH038
IMA1
Cu
IMA3
Mn
PYE
Zn
OPT3
Fe
FRO3
FRO7
ZIF1
YSL3
BTS
NAS3
ILR3
FER4
BHLH104
BHLH11
FER1

D

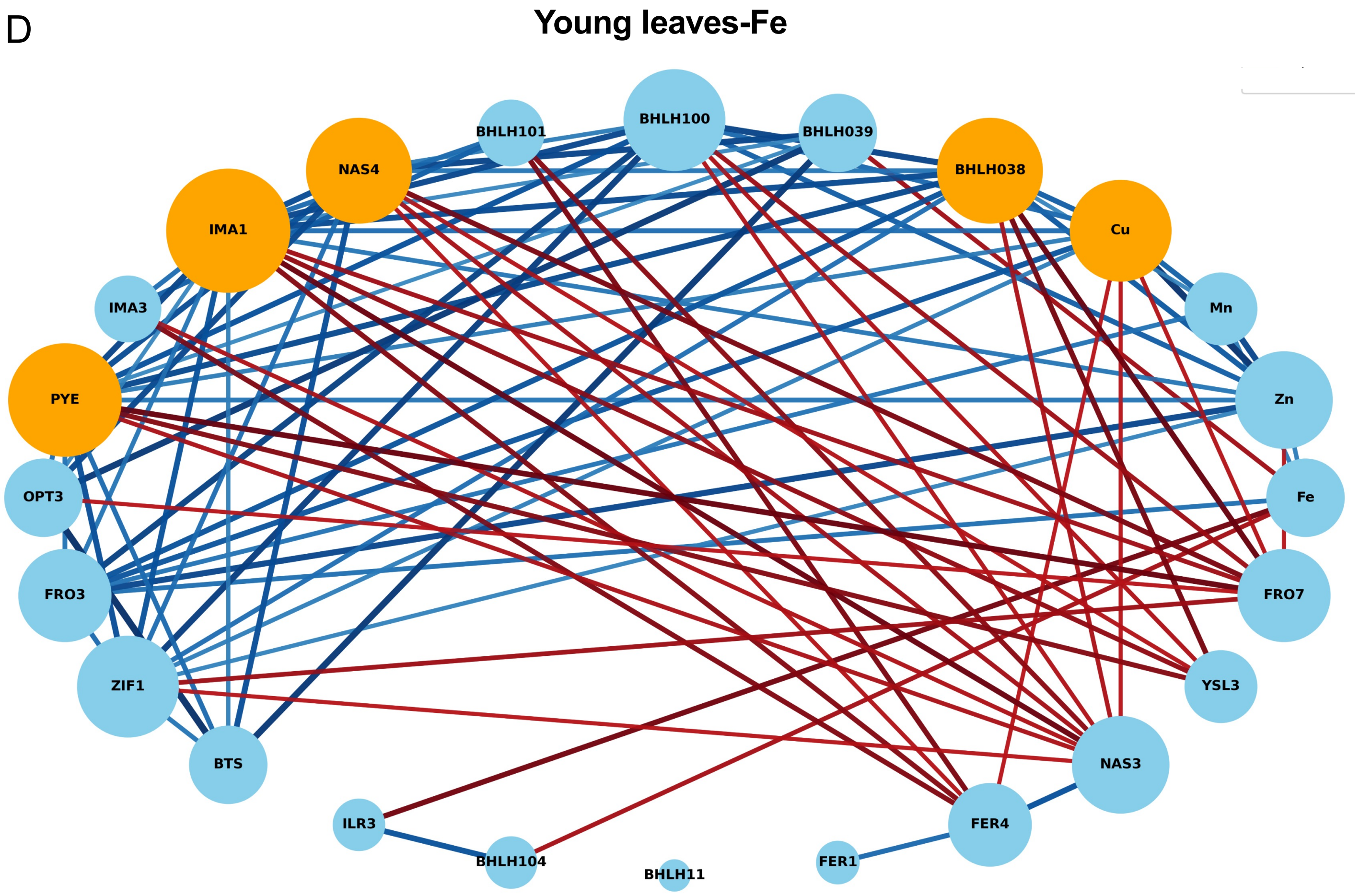
Young leaves-Fe
BHLH100
BHLH101
BHLH039
NAS4
BHLH038
IMA1
Cu
IMA3
Mn
PYE
Zn
OPT3
Fe
FRO3
FRO7
ZIF1
YSL3
BTS
NAS3
ILR3
FER4
BHLH104
BHLH11
FER1

Figure S7. Network analysis of correlations between genes and minerals Fe, Mn, Zn and Cu in old (A & B) and young leaves (C & D) at +Fe and –Fe conditions, respectively. Node sizes represent the degree centrality of each node, with larger nodes indicating genes or minerals with higher degree centrality while smaller nodes show genes with lower degree centrality. Orange nodes highlight most connected genes. Edge colors represent correlation, positive correlations are shown in blue and red for negative correlations. Edge thickness represent the strength of the correlation between connected nodes. Correlations that were above 0.5 are displayed, p value<0.05. Scale ranges from 1 to -1 to account for variation in correlation.
